# Platinum(II) Iodido Complexes of 7-Azaindoles with Significant Antiproliferative Effects: An Old Story Revisited with Unexpected Outcomes

**DOI:** 10.1371/journal.pone.0165062

**Published:** 2016-12-01

**Authors:** Pavel Štarha, Ján Vančo, Zdeněk Trávníček, Jan Hošek, Jarmila Klusáková, Zdeněk Dvořák

**Affiliations:** 1 Department of Inorganic Chemistry & Regional Centre of Advanced Technologies and Materials, Faculty of Science, Palacký University in Olomouc, Olomouc, Czech Republic; 2 Department of Human Pharmacology and Toxicology, Faculty of Pharmacy, University of Veterinary and Pharmaceutical Sciences Brno, Brno, Czech Republic; 3 Department of Cell Biology and Genetics & Regional Centre of Advanced Technologies and Materials, Department of Inorganic Chemistry, Faculty of Science, Palacký University in Olomouc, Olomouc, Czech Republic; Shiraz University, ISLAMIC REPUBLIC OF IRAN

## Abstract

A series of platinum(II) diiodido complexes containing 7-azaindole derivatives, having the general formula *cis*-[PtI_2_(*n*aza)_2_] (**1**–**8**), has been prepared and thoroughly characterized, including X-ray structure analysis of *cis*-[PtI_2_(*2Me4Cl*aza)_2_]∙DMF (**8**∙DMF; *2Me4Cl*aza = 2-methyl-4-chloro-7-azaindole). Complexes showed high *in vitro* cytotoxicity against nine human cancer cell lines (IC_50_ ranging from 0.4 to 12.8 μM), including the *cisplatin*-resistant ovarian cancer cell line (A2780R; IC_50_ = 1.0–3.5 μM). The results of *in vivo* testing, using the L1210 lymphocytic leukaemia model, at the equimolar doses of Pt with *cisplatin* (2 mg/kg) confirmed the activity of complex **8** comparable to *cisplatin*. From the mechanistic point of view, evaluated *ex vivo* by Western blot analyses on the samples of isolated tumour tissues, the treatment of the animals with complex **8**, contrary to *cisplatin*, decreased the levels of tumour suppressor p53 and increased significantly the amount of intracellular anti-apoptotic protein MCL-1_L_ (37 kDa). Additionally, the active form of caspase 3 was significantly elevated in the sample of tumour tissues treated with complex **8**, indicating that the activation of p53-independent cell-death pathway was initiated. The light and electron microscopy observations of the cancerous tissues revealed necrosis as a dominant mechanism of cell death, followed by scarce signs of apoptosis. The additional results (e.g. *in vitro* interaction experiments with selected biomolecules, cell cycle perturbations, gel electrophoretic studies on pUC19 plasmid DNA) supported the hypothesis that the complexes might be involved in the mechanism of action quite different from *cisplatin*.

## Introduction

Anticancer platinum(II) complexes are pharmaceutically successful group of compounds. To date, more than twenty representatives entered the clinical trials as the prospective candidates for the treatment of cancer [1]. Some of these complexes are currently used world-wide (*cisplatin*, *carboplatin*, *oxaliplatin*) or locally (*heptaplatin*, *nedaplatin*, *lobaplatin*) for the treatment of various types of cancer [1–2]. Interestingly, the clinically used, clinically studied and already discontinued platinum complexes are quite rigid in terms of their leaving groups. Most of complexes contain bidentate carboxylato ligands or some of them involve chlorido ligands as leaving groups. Only three complexes (*spiroplatin*, TRK-710 and *aroplatin*) contain the leaving groups different from the mentioned ones, in particular sulfato, 3-acetyl-5-methyl-2,4(3*H*,5*H*)-furandionato, and neodecanoato, respectively [1]. Nevertheless, the current knowledge of medicinal chemistry of the anticancer platinum complexes indicates that the scale of the applicable leaving groups could be larger. In this context, platinum(II) diiodido complexes seem to be prospective candidates.

Platinum(II) iodido complexes have long been overlooked by medicinal chemists, because several pioneer works reported them as inactive analogues of potent chlorido complexes (e.g. *cis*-[PtI_2_(NH_3_)_2_], an iodido analogue of *cisplatin*), thus declaring iodide as unsuitable ligand for development of novel platinum-based metallotherapeutics [3–4]. Remarkably, several complexes previously declared to be inactive, were more recently reported as antitumour active [5–6]. For example, *cis*-[PtI_2_(NH_3_)_2_] showed *in vitro* antitumour activity against various cancer cell lines including both *cisplatin*-sensitive (IC_50_ = 13.4 μM) and *cisplatin*-resistant (IC_50_ = 4.2 μM) HCT116 colon cancer cell lines (IC_50_ = 7.6, and 22.0 μM, respectively, for *cisplatin*) [6]. Apart from that, many other platinum(II) iodido complexes of various types (monofunctional [7–8], bifunctional [9–11], mixed-ligand [12–13] and multinuclear [14] complexes) showed considerable potency connected with different mechanism of action from those of the clinically used platinum-based drugs. For example, both isomers of [PtI_2_(*ip*am)_2_] (*ip*am = isopropylamine) showed high potency against a panel of human breast, cervix, non-small cell lung and colon carcinomas [9–10]. Moreover, these complexes showed different type of interaction with pBR322 plasmid DNA, more efficient induction of apoptosis as well as different cell cycle modification, as compared with *cisplatin*, indicating the mechanistic differences in relation to *cisplatin*. One of the reasons for the above said may be associated with the fact that *cis*-[PtI_2_(*ip*am)_2_] releases the amine ligands instead of iodido ones when interacting with *N*-acetyl-L-cysteine, 9-ethylguanine or single-stranded oligonucleotide [9–10]. A next example of the above-discussed may be documented by interaction of *cis*-[PtI_2_(NH_3_)_2_] with the a hen egg white lysozyme (HEWL), in which one of two NH_3_ ligands is released and the remaining [PtI_2_(NH_3_)] moiety forms a bond with the His15 imidazole residue in the structure of a HEWL protein, as suggested by X-ray crystallography [15]. To sum up, the above-cited studies [9,10,15] shed a new light on the topic of anticancer platinum iodido complexes, suggesting this type of compounds to be an interesting target for future development of new antitumour active complexes.

**Fig 1:**
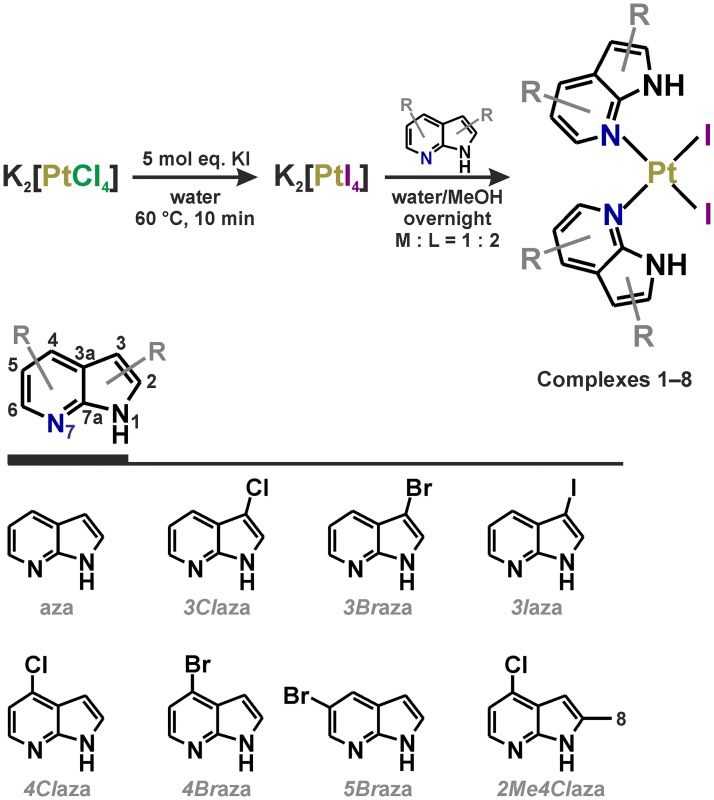
Synthetic pathway for the preparation of complexes 1−8. The structural formulas of the used 7-azaindole and its derivatives are also included.

With respect to these findings and to the promising results of anticancer screening of platinum(II) dichlorido complexes containing the 7-azaindole derivatives reported previously by our group [16–18] and others [19–20], we decided to study a series of *cis*-[PtI_2_(*n*aza)_2_] (**1**–**8**; Fig 1) complexes and further evaluate their *in vitro* and *in vivo* activities and mechanisms of action.

## Materials and Methods

### Chemicals

The chemicals K_2_[PtCl_4_] was purchased from Precious Metals Online, while KI, 7-azaindole, 3-chloro-7-azaindole, 3-bromo-7-azaindole, 3-iodo-7-azaindole, 4-chloro-7-azaindole, 4-bromo-7-azaindole, 5-bromo-7-azaindole, 4-chloro-2-methyl-7-azaindole, *cisplatin*, *oxaliplatin*, reduced glutathione, guanosine 5′-monophosphate disodium salt hydrate, ethidium bromide, tris(hydroxymethyl)aminomethane, calf thymus DNA, Roswell Park Memorial Institute medium, fetal calf serum, glutamine, penicillin and streptomycin, and solvents (methanol, diethyl ether, *n*-octanol, DMF-*d*_*7*_, D_2_O) were supplied by Sigma-Aldrich Co. and Fisher-Scientific Co.

### Synthesis

The platinum(II) diiodido complexes **1**–**8** (Fig 1) were prepared using a slight modification of the recently reported protocol [21]. Briefly, 0.5 mmol (207.5 mg) of K_2_[PtCl_4_] was dissolved in 5 mL of deionized water at 60°C and an excess of KI (415.0 mg; 2.5 mmol) was added to the red solution, which turned dark red over 10 min of stirring at 60°C. After cooling to ambient temperature, 1.0 mmol of *n*aza (118.1 mg for aza, 152.6 mg for *3Cl*aza and *4Cl*aza, 197.0 mg for *3Br*aza, *4Br*aza *5Br*aza, 244.0 mg for *3I*aza and 166.6 mg for *2Me4Cl*aza; Fig 1) dissolved in 5 mL of methanol was poured in. The mixture was stirred overnight at ambient temperature and the obtained yellow solid was removed by filtration and washed with deionized water (3 × 5 mL), methanol (3 × 2 mL) and diethyl ether (3 × 5 mL). The products (yields ~90%) were dried under vacuum and stored in desiccator over silica gel.

*cis*-[Pt(aza)_2_I_2_] (**1**): ^1^H NMR (400 MHz, DMF-*d*_*7*_, 300 K): δ 13.33 and 12.78 (br, N1H, 1H), 8.97 (d, 1H, *J =* 4.7 Hz, C6H), 8.03 (br, 1H, C4H), 7.80 (s, 1H, C2H), 7.20 (br, 1H, C5H), 6.60 (s, 1H, C3H) ppm. ^13^C NMR (100 MHz, DMF-*d*_*7*_, 300 K): δ 148.6 (C7a), 145.9 (C6), 132.2 (C4), 128.6 (C2), 124.3 (C3a), 117.7 (C5), 102.8 (C3) ppm. Anal. Calcd. for C_14_H_12_N_4_I_2_Pt: C, 24.54; H, 1.77; N, 8.18. Found: 24.40; H, 1.63; N, 8.02%.

*cis*-[Pt(*3Cl*aza)_2_I_2_] (**2**): ^1^H NMR (400 MHz, DMF-*d*_*7*_, 300 K): δ 13.76 and 13.07 (br, 1H, N1H), 9.10 (d, 1H, *J* = 5.3 Hz, C6H), 8.08 (br, 1H, C4H), 8.03 (s, 1H, C2H), 7.37 (m, 1H, C5H) ppm. ^13^C NMR (100 MHz, DMF-*d*_*7*_, 300 K): δ 147.5 (C6), 146.2 (C7a), 130.0 (C4), 126.2 (C2), 122.1 (C3a), 118.4 (C5), 105.0 (C3) ppm. Anal. Calcd. for C_14_H_10_N_4_Cl_2_I_2_Pt: C, 22.30; H, 1.34; N, 7.43. Found: 22.60; H, 1.27; N, 7.57%.

*cis*-[Pt(*3Br*aza)_2_I_2_] (**3**): ^1^H NMR (400 MHz, DMF-*d*_*7*_, 300 K): δ 13.87 and 13.17 (br, 1H, N1H), 9.11 (br, 1H, C6H), 8.11 (br, 1H, C2H), 7.99 (s, 1H, C4H), 7.34 (br, 1H, C5H) ppm. ^13^C NMR (100 MHz, DMF-*d*_*7*_, 300 K): δ 147.4 (C6), 146.1 (C7a), 130.8 (C4), 128.8 (C2), 123.6 (C3a), 118.9 (C5), 90.0 (C3) ppm. Anal. Calcd. for C_14_H_10_N_4_Br_2_I_2_Pt: C, 19.95; H, 1.20; N, 6.65. Found: 19.72; H, 1.12; N, 6.43%.

*cis*-[PtI_2_(*3I*aza)_2_] (**4**): ^1^H NMR (400 MHz, DMF-*d*_*7*_, 300 K): δ 13.85 and 13.19 (br, 1H, N1H), 9.06 (br, 1H, C6H), 8.09 (br, 1H, C2H), 7.79 (s, 1H, C4H), 7.33 (br, 1H, C5H) ppm. ^13^C NMR (100 MHz, DMF-*d*_*7*_, 300 K): δ 147.2 (C7a), 145.8 (C6), 133.6 (C2), 132.4 (C4), 126.7 (C3a), 118.8 (C5), 56.4 (C3) ppm. Anal. Calcd. for C_14_H_10_N_4_I_4_Pt: C, 17.95; H, 1.08; N, 5.98. Found: 17.53; H, 1.02; N, 5.49%.

*cis*-[Pt(*4Cl*aza)_2_I_2_] (**5**): ^1^H NMR (400 MHz, DMF-*d*_*7*_, 300 K): δ 13.77 and 13.12 (br, 1H, N1H), 8.98 (br, 1H, C6H), 7.95 (br, 1H, C2H), 7.37 (br, 1H, C5H), 6.67 (br, 1H, C3H) ppm. ^13^C NMR (100 MHz, DMF-*d*_*7*_, 300 K): δ 148.0 (C7a), 146.4 (C6), 138.4 (C4), 129.6 (C2), 123.3 (C3a), 118.0 (C5), 101.1 (C3) ppm. Anal. Calcd. for C_14_H_10_N_4_Cl_2_I_2_Pt: C, 22.30; H, 1.34; N, 7.43. Found: 22.07; H, 1.46; N, 6.85%.

*cis*-[Pt(*4Br*aza)_2_I_2_] (**6**): ^1^H NMR (400 MHz, DMF-*d*_*7*_, 300 K): δ 13.78 and 13.12 (br, 1H, N1H), 8.88 (br, 1H, C6H), 7.96 (br, 1H, C2H), 7.51 (br, 1H, C5H), 6.59 (br, 1H, C3H) ppm. ^13^C NMR (100 MHz, DMF-*d*_*7*_, 300 K): δ 147.0 (C7a), 146.0 (C6), 129.6 (C2), 128.0 (C3a), 125.7 (C4), 121.1 (C5), 102.7 (C3) ppm. Anal. Calcd. for C_14_H_10_N_4_Br_2_I_2_Pt: C, 19.95; H, 1.20; N, 6.65. Found: 19.60; H, 1.22; N, 6.25%.

*cis*-[Pt(*5Br*aza)_2_I_2_] (**7**): ^1^H NMR (400 MHz, DMF-*d*_*7*_, 300 K): δ 13.66 and 12.97 (br, 1H, N1H), 9.26 (br, 1H, C6H), 8.27 (br, 1H, C4H), 7.90 (br, 1H, C2H), 6.60 (br, 1H, C3H) ppm. ^13^C NMR (100 MHz, DMF-*d*_*7*_, 300 K): δ 146.8 (C7a), 145.8 (C6), 134.4 (C4), 130.8 (C2), 126.0 (C3a), 110.9 (C5), 102.7 (C3) ppm. Anal. Calcd. for C_14_H_10_N_4_Br_2_I_2_Pt: C, 19.95; H, 1.20; N, 6.65. Found: 19.55; H, 1.03; N, 6.23%.

*cis*-[PtI_2_(*2Me4Cl*aza)_2_] (**8**): ^1^H NMR (400 MHz, DMF-*d*_*7*_, 300 K): δ 13.52 and 12.84 (br, 1H, N1H), 8.83 (s, 1H, C6H), 7.30 (s, 1H, C5H), 6.37 (s, 1H, C3H), 2.63 (s, 3H, C8H) ppm. ^13^C NMR (100 MHz, DMF-*d*_*7*_, 300 K): δ 148.4 (C7a), 143.6 (C6), 141.4 (C4), 136.6 (C2), 124.0 (C3a), 118.2 (C5), 98.8 (C3), 14.1 (C8). Anal. Calcd. for C_16_H_14_N_4_Cl_2_PtI_2_: C, 24.57; H, 1.80; N, 7.16. Found: 24.32; H, 1.72; N, 7.04%.

### General Methods

^1^H and ^13^C NMR spectroscopy and ^1^H–^1^H gs-COSY, ^1^H–^13^C gs-HMQC and ^1^H–^13^C gs-HMBC two dimensional correlation experiments (gs = gradient selected, COSY = correlation spectroscopy, HMQC = heteronuclear multiple quantum coherence, HMBC = heteronuclear multiple bond coherence) of the DMF-*d*_*7*_ solutions were performed at 300 K on either Varian 400 device (at 400.0 MHz (^1^H) or 100.6 MHz (^13^C)) or JEOL JNM-ECA 600II device (at 600.00 MHz (^1^H) or 150.86 MHz (^13^C)). ^1^H and ^13^C NMR spectra were calibrated against the residual DMF-*d*_*6*_^1^H NMR (8.03, 2.92 and 2.75 ppm) and ^13^C NMR (163.2, 34.9 and 29.8 ppm) signals. The splitting of proton resonances in the reported ^1^H spectra is defined as s = singlet, d = doublet, t = triplet, br = broad band, m = multiplet. Electrospray ionization mass spectrometry (ESI-MS) was performed by LCQ Fleet ion trap spectrometer (Thermo Scientific; QualBrowser software, version 2.0.7) in positive (ESI+) and negative (ESI–) ionization modes of the methanol solutions. Elemental analyses were carried out using Flash 2000 CHNS Elemental Analyzer (Thermo Scientific).

### X-ray Crystallography

Single crystals of *cis*-[PtI_2_(*2Me4Cl*aza)_2_]∙DMF (**8**∙DMF) were grown from the saturated DMF solution of *cis*-[PtI_2_(*2Me4Cl*aza)_2_] by a diffusion method with diethyl ether. A suitable crystal was selected and placed on an Xcalibur^™^2 diffractometer (Oxford Diffraction Ltd.) equipped with a Sapphire2 CCD detector, using Mo Kα radiation (monochromator Enhance, Oxford Diffraction Ltd.) and ω-scan rotation techniques. The crystal was kept at 120(2) K during data collection. The CRYSALIS software package (Oxford Diffraction Ltd.) was used for data collection and reduction [22]. The structure was solved with the *ShelX-*97 [23] structure solution programs using direct methods and refined on *F*^2^ using a full-matrix weighted least-squares procedure. All H-atoms were found from difference Fourier maps and refined using a riding model with AFIX 43 and AFIX 137 instructions, with C–H = 0.95 Å for (CH)_aromatic_, 0.98 Å for (CH_3_), and N–H = 0.88 Å for (NH), and with *U*_iso_(H) = 1.2 *U*_eq_(CH, NH) and 1.5 *U*_eq_(CH_3_). X-ray crystallographic data for complex **8**∙DMF have been deposited in the Cambridge Crystallographic Data Centre under the accession number CCDC 1480147. The crystal data and structure refinements are given in S1 Table and the Crystallographic Information File is available for download as S1 File. The graphics were drawn and additional structural calculations were performed by DIAMOND [24] and Mercury [25] software.

### Studies of Solution Chemistry and Interactions with Biomolecules

Complex **6** was dissolved in 120 μL of DMF-*d*_*7*_ and diluted with 480 μL of D_2_O to give the solution of ca 1 mM concentration; a presence of DMF ensured the solubility of complex **6**, with respect to its low solubility in water. Similar solutions were prepared with two molar equivalents of either GSH or GMP dissolved in 480 μL of D_2_O subsequently added into the solution of complex **6** dissolved in 120 μL of DMF-*d*_*7*_. ^1^H NMR spectra were recorded at different time points over 48 h at 300 K using a JEOL JNM-ECA 600II device. The obtained ^1^H NMR spectra were calibrated against the residual signal of DMF-*d*_*6*_ found at 8.03 ppm. Similar experiments were carried out also in non-deuterated solvents and evaluated by ESI+ mass spectrometry.

### Cell Cultures

A2780 ovarian carcinoma, A2780R *cisplatin*-resistant ovarian carcinoma, HOS osteosarcoma, G361 malignant melanoma, MCF7 breast carcinoma, A549 lung carcinoma, HeLa cervix carcinoma, 22Rv1 prostate carcinoma and Caco-2 colorectal carcinoma human cancer cell lines were purchased from the European Collection of Cell Cultures (ECACC), while the human non-cancerous hepatocytes (Hep) were supplied by Biopredic Intl. The cell lines were cultured, according to the suppliers`instructions, in RPMI-1640 medium supplemented with 10% of fetal calf serum, 1% of 2 mM glutamine, and 1% penicillin/streptomycin. All cell lines were grown as adherent monolayers at 37°C and 5% CO_2_ in a humidified atmosphere.

### *In Vitro* Cytotoxicity Testing

Appropriate amount of the tested compounds (**1**–**8**, *cisplatin* and *oxaliplatin*) was dissolved in DMF to give the 50 mM stock solutions.

The cells were seeded to 96-well culture plates, pre-incubated in drug-free media at 37°C for 24 h and treated typically with the 0.01–50.0 μM solutions, prepared from the stock solutions by dilution with medium, for 24 h at 37°C. The Caco-2 cells and hepatocytes were treated by *cisplatin* up to higher than 50.0 μM concentrations, in particular 90.0 and 75.0 μM, respectively. *Cisplatin* was used as the reference drug for all the cell lines used, while *oxaliplatin* served as the standard only in the case of Caco-2 cells. The highest applicable concentration of *oxaliplatin* (25.0 μM) is given by its limited solubility in the medium used. In parallel with compounds **1**–**8** and the reference drugs, the cells were also treated with vehicle (0.1% DMF in medium; negative control) and Triton X-100 (1%; positive control) to assess the minimal and maximal cell damage, respectively. The MTT assay was used to determine the cell viability; MTT = 3-(4,5-dimethylthiazol-2-yl)-2,5-diphenyltetrazolium bromide. A concentration of the formed dye was evaluated spectrophotometrically at 540 nm (TECAN, Schoeller Instruments LLC). Complexes **5** and **6** (and *cisplatin* for comparative purposes) were also tested for their time-dependent *in vitro* cytotoxicity against A2780 cancer cell line at different time points (6 h, 24 h, 48 h).

The data were expressed as the percentage of viability, where 100% and 0% represents the treatments with negative and positive controls, respectively. The data from the cancer cells were acquired from three independent experiments (conducted in triplicate) using cells from different passages. The resulting IC_50_ values (μM) were calculated from viability curves and the results are presented as arithmetic mean±SD.

### Hydrophobicity Studies (*logP* Determination)

Octanol-saturated water (OSW) and water-saturated octanol (WSO) were prepared from octanol and 0.2 M KI solution in distilled water (overnight shaking; Vibramax 100, Heidolph Instruments). Complexes **4** and **6** (1 mmol) were ultrasonicated for 15 min in 10 mL of OSW, centrifuged and supernatants were collected. Aliquots (5 mL) of the obtained OSW solutions of complexes **4** and **6** were added to WSO (5 mL) and shaken for 2 h at ambient temperature. After that, the mixtures were centrifuged and aqueous layer was separated carefully. The platinum concentration was determined from OSW aliquots taken before ([Pt]OSW_b_) and after ([Pt]OSW_a_) partition by ICP-MS (ICP-MS spectrometer 7700x, Agilent) with the obtained values corrected for the adsorption effects. *logP* = log([Pt]WSO/[Pt]OSW_a_) equation was used for the partition coefficients calculation; [Pt]WSO = [Pt]OSW_b_−[Pt]OSW_a_. The experiments were conducted in triplicate.

### Cellular Accumulation

The A2780 cells were seeded in 6-well culture plates (1×10^6^ cells per well) and incubated overnight (37°C and 5% CO_2_ in a humidified incubator). After that the cells were treated with the IC_50_ concentrations of complexes **4**, **6** and *cisplatin*. The cells were washed with PBS (2 × 2 mL) after 24 h treatment, harvested by trypsin treatment, collected and centrifuged in PBS. The supernatants were discarded after centrifugation and pellets were digested in 500 μL of nitric acid (3 min at 150°C) using a microwave system Monowave300 (Anton Paar). Solutions were diluted with 4.5 mL of water and the platinum content was determined by ICP-MS. The obtained values were corrected for adsorption effects. The experiments were conducted in triplicate and the results are presented as arithmetic mean±SD.

### Cell Cycle Analysis

The A2780 and MCF7 cells (1.0 × 10^6^ per well) were pre-incubated in a six-well plate for 24 h as described above. The selected complexes **6** and **8** were added at the concentrations equal to IC_50_ (*cisplatin* was involved in the study for comparative purposes). After 24 h, floating cells were collected and attached cells were harvested using trypsin/EDTA in PBS. Total cells were washed twice with PBS and fixed in 70% ethanol. Cells were re-suspended in PBS and DNA staining was achieved by a solution of propidium iodide (PI) supplemented with RNase A (30 min, 25°C, in the dark). After that, the cells were washed (PBS), re-suspended (PBS) and DNA content was measured using flow cytometry (CytoFlex, Beckman Coulter) detecting emission of DNA-bound PI (maximum at 617 nm) after excitation at 535 nm. The data were analysed using CytExpert^™^ software (Beckman Coulter).

### Fluorescence Quenching Experiments

A volume of 405 μL of 154 μM ctDNA and 10 μL of 3 mM EtBr were mixed together in TRIS/NaCl buffer (pH = 7.2) and incubated for 30 min at ambient temperature. 0, 100, 200, 400, 600 and 1000 μL of 150 μM *4Br*aza, *2Me4Cl*aza or representative complexes **6** and **8**, and *cisplatin* for comparative purposes (corresponding to the final concentration of 0, 5.0, 10.0, 20.0, 30.0 and 50.0 μM) dissolved in 10% methanol solution in TRIS/NaCl buffer were added to the pre-incubated EtBr/ctDNA system and the volume was adjusted to 3 mL with TRIS/NaCl buffer. The mixtures were incubated at ambient temperature for next 15 min. The maxima of the emitted fluorescence of EtBr/ctDNA system have been observed at 613 nm (using the 546 nm excitation wavelength) and the intensity of fluorescence was recorded on a AvaSpec HS1024x122TE fluorescence spectrometer using a 1 cm quartz cell. The Stern-Volmer quenching constants, *K*_SV_ (M^–1^), were calculated according the equation *F*_0_/*F* = 1 + *K*_SV_∙[Q]; *F*_0_ and *F* stand for EtBr/DNA fluorescence intensity in the absence and presence of the complex, respectively, and [Q] is the molar concentration of the quencher, i.e. the concentration of complex **6**, complex **8** or *cisplatin* [26]. Due to the indirect nature of the fluorescence quenching method, the value of *K*_SV_ can be used for the estimation of the apparent binding constant *K*_app_ of the complex by using the equation *K*_EB_ [EtBr] = *K*_app_ [Q_50_], where [Q_50_] is the concentration of the complex causing the 50% reduction in the intensity of fluorescence of the EtBr/DNA supramolecular complex, *K*_EB_ = 1.0 × 10^6^ M^−1^ and [EtBr] = 10 μM [27].

### Gel Electrophoresis Studies of Interaction with pUC19 Plasmid dsDNA

Supercoiled pUC19 plasmid dsDNA (2686 bp, 1750 kDa) was isolated from *Escherichia coli* TOP10F’ by QIAprep Spin Miniprep Kit (Qiagen, Germany). The concentration and purity of the isolated plasmid DNA were evaluated spectrophotometrically. Plasmid was dissolved in nuclease-free water and stored at –20°C.

Stock solutions of complexes **6** and **8**, and *cisplatin* were prepared in three different concentrations (23.0, 11.5 and 2.30 μM) in DMF. 2 μL of these solutions were added to 18 μL of the pUC19 plasmid DNA water solution (300 ng per reaction, corresponding to a concentration of 23 μM calculated for the base pairs) and the mixture was left to react in the final 10% DMF/90% water solution containing the final concentrations of the complexes of 2.30, 1.15 and 0.23 μM, corresponding to 0.10, 0.05 and 0.01 metal-to-base pair stoichiometry. In parallel, the experiment was performed on plasmid DNA in the 10% DMF/90% water solution and in water. The reaction mixtures were incubated at 37°C for 24 h. An electrophoretic mobility was evaluated by gel electrophoresis (90 V/cm, 3 h) performed on a 0.8% (w/v) agarose gel containing EtBr (2.0 mg/L (w/v)) at ambient temperature in Tris-acetate/EDTA buffer. The bands on the obtained electrophoretogram were detected by an MUV21-CP-02 gel scanner (Major Science) and analysed with AlphaEaseFC software (version 4.0.0.34, Alpha Innotech) in order to evaluate the relative percentages of the SC, OC and L forms. Each experiment was performed in triplicate.

### Animals

The experimental animals used for *in vivo* antitumour testing were female DBA/2 SPF mice obtained from Charles Rivers Laboratories. The animals were housed in the Sealsafe NEXT—IVC Blue Line Housing System (Tecniplast, Italy) to ensure the best possible experimental conditions and eliminate the risk of possible intergroup cross-contamination, and kept in Plexiglas cages at constant temperature (22±1°C) and relative humidity of 55±5% for one-week adaptation period before the start of the experiment. All experimental procedures were performed according to the National Institute of Health (NIH) Guide for the Care and Use of Laboratory Animals. The protocol was approved by the Expert Committee on the Protection of Animals Against Cruelty at the University of Veterinary and Pharmaceutical Sciences Brno (Permit Number: 71/2013). The number of pharmacological interventions was limited to the necessary minimum in order to minimize the suffering of animals, which were observed regularly for the symptoms connected with the tumour progression. The criteria set for the humane endpoints during the study were as follows: 1/ the loss of >25% body weight as compared with the initial values (measured regularly each day in the morning); 2/ acute toxicity symptoms (e.g. tremors, paralysis, etc., observed regularly every 2 hours); and 3/ excessive volume of the tumour tissues hindering the free movement of the animals (observed regularly every 2 hours). No animals had to be sacrificed due to the above mentioned criteria of humane treatment and no post-application deaths within the 2 hours after the application were observed during the experiment. All unexpected deaths (i.e. the animals were found dead) were proved by the histological examination to be caused by the excessive tumour progression. The animal tissues for the *ex vivo* experiments and histological evaluations were taken *post mortem*, in case of surviving animals immediately after they were sacrificed by the lethal dose of carbon dioxide inhalation, immediately followed by the cervical dislocation.

### *In Vivo* Antitumour Activity Testing

After one-week adaptation, animals were randomly assigned to five groups (*n* = 10)—one control group (received 10% DMF in water (v/v)) and four groups of animals treated with complexes **5**, **6** and **8**, and the reference drug *cisplatin*. Complexes (including *cisplatin*) were dissolved in DMF for molecular biology and diluted with sterile isotonic saline solution to the final content of 10% DMF. Due to the lack of acute toxicity data and in order to eliminate the excessive use of laboratory animals, the doses of complexes **5**, **6** and **8** equimolar to maximum tolerated dose of *cisplatin* (2 mg/kg) were applied intraperitoneally 10 days after the intraperitoneal implantation of 10^6^ L1210 mouse lymphocytic leukaemia cells. The animals were monitored several times a day for the signs of tumour progression and distress development. The experimental data were collected for all groups of animals as mean survival time (days), and further expressed as the percentage of mean survival time extension, T/C (%), defined as the ratio of the mean survival time of the animals treated either with complexes **5**, **6** and **8** or *cisplatin* (T) and the control group animals (C).

### Histological Observations

The samples of different tissues, dominantly spleen, renal tissue including the perirenal fat and half of the isolated diffuse and well-circumcised tumours, from *in vivo* antitumour activity testing were fixed in 10% neutral buffered formaldehyde, dehydrated by an increasing amount of alcohol (30%–50%–70%–80%–95%–100%), cleared in xylene and embedded in paraffin. Randomly selected paraffin block preparations were stained by standard hematoxylin and eosin stain for light microscopic evaluation. Due to high cellularity, the semi-quantitative method was used for the evaluation of necrosis in well-circumcised and diffuse tumour samples and PMN cells infiltration in normal tissues as an indirect sign of toxicity to healthy tissues. The evaluation of necrotic processes and infiltration by PMN cells was semi-quantitative, based on the percentage of necrotic areas and/or infiltrated area in the view-field of at least three different samples. The scale from 0 to 4 was used, where 0 = without necrosis, 1 = up to 25%, 2 = up to 50%, 3 = up to 75% and 4 = up to 100% of the areas in the view field.

Randomly selected sections from the tumour tissues (from the control group and the groups treated with *cisplatin* and complex **5**) were further processed for transition electron microscopy observations of cellular and subcellular processes. The samples in paraffin blocks were deparaffinised, transferred into acetone and rehydrated by an increasing amount of water (100%–90%–75%–50%–25%), each step for 15 min. The samples were rinsed with phosphate buffered solution (pH 7.4), fixed with glutaraldehyde for 1 h and after that contrasted with osmium tetroxide solution for 2 h. In continuation, the samples were dehydrated once again by an increasing amount of acetone (25%–50%–75%–90%–100%) and the dehydrated samples were saturated with Durcupan and Epon and transferred to gelatine capsules for polymerization at 56°C for 3 days. The embedded blocks were cut to 90–150 nm thick sections using a ultramicrotome and subsequently contrasted with uranyl acetate solution for 30 min and Reynolds solution for 10 min. The sections were observed using the Tescan VEGA3 LM scanning electron microscope equipped with the scanning transmission electron microscopy (STEM) detector in both Bright Field and Dark Field at magnification of ca 50,000×. The morphological changes in the tissue and cell integrity and cell organelles (if possible) were observed.

### Protein Expression Analysis

Frozen tumour samples were homogenized by a bench blender IKA DI25 (IKA-Werke, Germany) in the presence of a lysis buffer [cOmplete Protease Inhibitor Cocktail (Roche, Germany), 50 mM Tris-HCl pH 7.5, 1 mM EGTA, 1 mM EDTA, 1 mM sodium orthovanadate, 50 mM sodium fluoride, 5 mM sodium pyrophosphate, 270 mM sucrose, 0.5% (v/v) Triton X-100]. Subsequently, the blended samples were centrifuged at 9.000 g at 4°C for 15 min. The supernatants were collected and the protein concentration was measured by the Brandford’s method using the Brandford reagent (Amresco, USA) according to the manufacturer’s manual. The measured samples were stored at -80°C for the following experiments.

The expression of caspase 3, MCL-1, p53, and β-actin as loading control were evaluated by Western blot analysis and immunodetection. Lysates were mixed with a denaturing loading dye [250 mM Tris-HCl pH 6.8, 5% (v/v) β-mercaptoethanol, 10% (w/v) sodium dodecyl sulfate, 30% (v/v) glycerol, 0.04% (w/v) bromophenol blue], heated for 5 min at 70°C. To eliminate intra-species variability, and to obtain the same amount of proteins from all experimental groups, all samples from one group of animals were pooled together and loaded onto a 15% polyacrylamide denaturing gel. The final protein amount was 50 μg per well. After protein separation in the gel, they were transferred on the Immun-Blot polyvinylidene difluoride membrane 0.2 μm (Bio-Rad, USA). Then, the membrane was blocked by 5% (w/v) BSA in TBST [10 mM Tris-HCl pH 7.5, 150 mM NaCl, 0.1% (v/v) Tween-20]. The following primary and secondary antibodies [conjugated with horseradish peroxidase (HRP)] were used for immunodetection: cleaved caspase 3 (Cell Signaling, USA; product number 9664) in the dilution of 1:2500, MCL-1 (Abcam, UK; product number ab32087) in the dilution of 1:2000, p53 (Abcam, UK; product number ab26) in the dilution of 1:2000, and β-actin (Abcam, UK; product number ab8226) in the dilution of 1:5000, goat anti-mouse antibody in the dilution of 1:2500 (Sigma-Aldrich, USA; product number A0168), goat anti-rabbit antibody in the dilution of 1:2500 (Sigma-Aldich, USA; product number A0545). HRP was detected by Clarity Western ECL Substrate (Bio-Rad, USA) using chemiluminescent documentation system Syngene PXi-4 (UK).

### Statistical Analysis

An ANOVA test was used for statistical analysis with the values of *p* < 0.05 (*), 0.01 (**) and 0.005 (***) considered to be statistically significant. QC Expert 3.2 Statistical software (TriloByte Ltd.) was used to perform the analysis.

## Results and Discussion

### Chemistry

Complexes **1**–**8** were prepared following the well-established Dhara´s method [28] using K_2_[PtI_4_] as a key intermediate (Fig 1). The compositions of complexes **1**–**8** were proved by ^1^H and ^13^C NMR spectroscopy, which have been performed for all the studied complexes together with 2D experiments (S1 Text; see Experimental Section) necessary for the relevant assignment of the appropriate ^1^H and ^13^C NMR signals. The ^1^H NMR spectra showed five (for **1** containing unsubstituted aza), four (for **2**–**7** containing monosubstituted *n*aza) or three (for **8** containing disubstituted *2Me4Cl*aza) signals of aromatic C–H hydrogens between 6.4–9.3 ppm. A CH_3_ singlet of *2Me4Cl*aza was detected at 2.63 ppm in the ^1^H NMR spectrum of complex **8**. Regarding the ^13^C NMR spectra, the chemical shift values are dependent on the substitution of the corresponding or adjacent carbon atoms. The most evident changes were observed for C3, whose δ was found to be 101.1–102.8 ppm for 7-azaindole and its C4- and C5-derivatives. However, in the case that the C3 atom is substituted by Cl (*3Cl*aza in **2**), Br (*3Br*aza in **3**) or I (*3I*aza in **4**), its chemical shift changed markedly and equalled 105.0, 90.0, and even 56.4 ppm, respectively. Similar situation can be discussed also for the C4 atom. The chemical shift changes between the free *n*aza ligands and the corresponding complexes, known as the coordination shifts (Δδ = δ_complex_− δ_ligand_; ppm), were also analysed, and the highest ^1^H NMR Δδ was observed for C6H adjacent to the *N*7 coordination site (0.70–0.96 ppm), while the coordination shifts of other C–H hydrogen atoms were found to be 0.05–0.33 ppm (S2 Table).

The ESI+ mass spectra of complexes **1**–**8** contained the molecular peaks of {[PtI_2_(*n*aza)_2_]+A}^+^ (A = H^+^, Na^+^ or K^+^), while the ESI–mass spectra revealed the pseudomolecular peaks corresponding to {[PtI_2_(*n*aza)_2_]–H}^−^(S2 Text, S1 Fig). The results of RP-HPLC/MS experiments (performed for the representative complexes **6** and **8**) showed one dominant HPLC peak at *t*_R_ = 2.39 min for complex **6** and *t*_R_ = 3.14 min for complex **8**, corresponding to the assumed composition of the complexes and proving their >99% purity. Higher *t*_R_ of complex **8** determined by RP-HPLC/MS suggested its higher relative lipophilicity than for complex **6** [29–30].

The representative complex **6**, chosen for its high *in vitro* cytotoxicity (see below) and a relatively good solubility, was highly hydrolytically stable in a mixture of 20% DMF-*d*_*7*_/80% D_2_O (the presence of DMF ensured the sufficient solubility of **6**), because only ca 10% of complex **6** hydrolyzed after 48 h (S2 Fig). Complex **6** did interact with reduced glutathione (GSH) in 20% DMF-*d*_*7*_/80% D_2_O (^1^H NMR and ESI+ MS studies; S3 Fig). Similarly, complex **6** was found to be inert to GMP, in contrast to *cisplatin*, which exhibited reactivity toward GMP under the used experimental conditions (20% DMF-*d*_*7*_/80% D_2_O) (S4 Fig).

### X-ray Structure of Complex 8∙DMF

**Fig 2:**
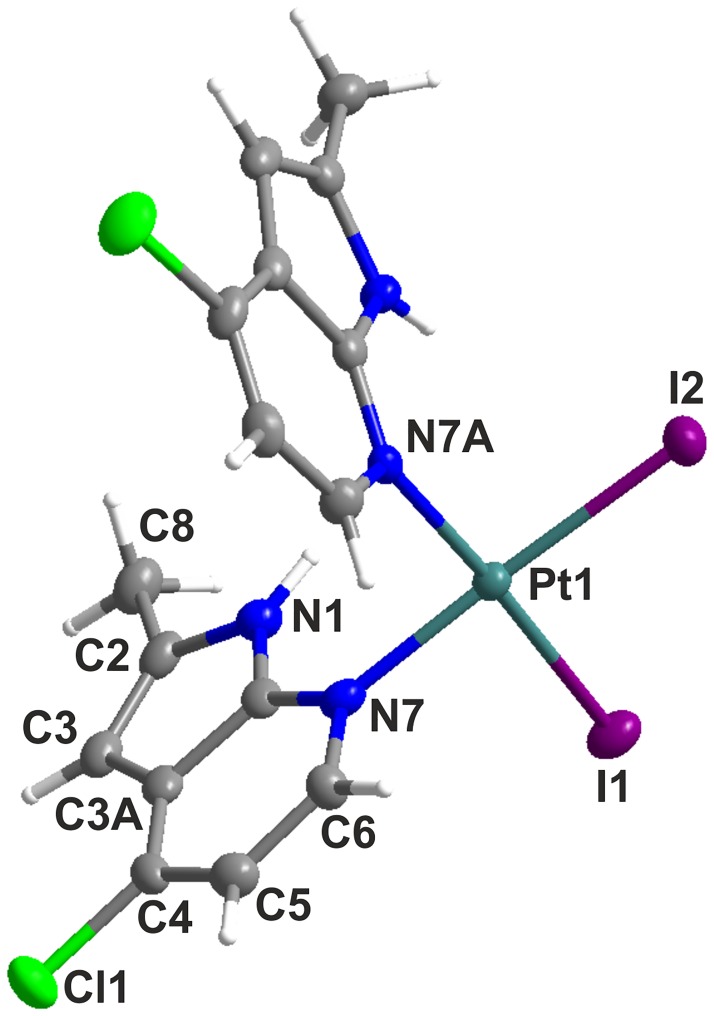
X-ray molecular structure of *cis*-[PtI_2_(*2Me4Cl*aza)_2_]∙DMF (8∙DMF). DMF molecule of crystallization is omitted for clarity, thermal ellipsoids are drawn at the 50% probability level.

The central Pt(II) atom of *cis*-[PtI_2_(*2Me4Cl*aza)_2_]∙DMF (**8**∙DMF) reveals a distorted square-planar geometry with two iodido ligands and two monodentate-coordinated *2Me4Cl*aza molecules mutually arranged in a *cis*-position (Fig 2, Table 1 and S1 Table). In the molecular structure of complex **8**, the two *2Me4Cl*aza ligands are head-to-head oriented and form a dihedral angle of 88.98(11)° (the least-squares planes fitted through the non-hydrogen atoms of the 7-azaindole rings). The bond lengths between the Pt(II) atom and donor atoms (I1, I2, N7 and N7A; Table 1) are consistent with those found in CCDC database for seventeen square-planar platinum(II) diiodido complexes, containing two heterocyclic *N*-donor ligands, whose values equal to 1.985–2.073 Å for Pt–N (average of 2.033 Å) and 2.575–2.616 Å for Pt–I (average of 2.597 Å). On the other hand, the Pt–N bond lengths are shorter for complex **8**∙DMF (Table 1) than those in dichlorido (2.023(2) and 2.009(2) Å for *cis*-[Pt(aza)_2_Cl_2_]∙DMF, and 2.020(3) and 2.012(3) Å for *cis*-[PtCl_2_(*3Cl*aza)_2_]∙DMF) or oxalato (2.001(4) and 2.009(4) Å for [Pt(aza)_2_(ox)]) complexes [16,31]. Similarly, different Pt–N bond lengths have been also observed for *cis*-[PtI_2_(4pic)_2_] (Pt–N = 2.056(13) and 2.060(14) Å) and *cis*-[PtCl_2_(4pic)_2_] (Pt–N = 2.005(4) and 2.017(5) Å) complexes containing 4-methylpyridine (4pic) [32–33].

**Table 1:** Selected bond lengths (Å) and angles (°) for *cis*-[PtI_2_(*2Me4Cl*aza)_2_]∙DMF (8∙DMF).

Bond lengths (Å)		Bond angles (°)	
Pt1−N7	2.044(4)	N7–Pt1–N7A	88.9(2)
Pt1–N7A	2.055(4)	I1–Pt1–I2	92.237(13)
Pt1–I1	2.5833(4)		
Pt1–I2	2.5836(4)		
		N7–Pt1–I2	175.97(12)
		N7A–Pt1–I1	176.68(11)

The crystal structure of complex **8**∙DMF is stabilized by the N–H⋯O hydrogen bonds between the N1 atoms of the *2Me4Cl*aza molecules and oxygen atoms of the DMF molecules of crystallization (N1⋯O1, N1A⋯O1; S5 Fig), and C–H⋯C, C–H⋯I and C⋯Cl types of non-covalent contacts (S6 Fig and S3 Table). Two individual molecules of the complex are mutually connected through the C2A⋯Cl1^i^ and C3A⋯Cl1^i^ contacts, thus forming the centrosymmetric dimers (S6 Fig; symmetry code: i) 1-x, 1-y, 1-z).

### *In Vitro* Antitumour Activity

*In vitro* cytotoxicity of complexes **1**–**8** was evaluated against the panel of nine human cancer cell lines (i.e. HOS, G361, MCF7, A549, HeLa, 22Rv1, A2780, A2780R and Caco-2). The selectivity of the compounds was assessed against normal cell culture of human hepatocytes (Hep). *Cisplatin* (for all the used cell lines) and *oxaliplatin* (only for Caco-2 cell line) were used for comparative purposes. The results are summarized in Table 2.

**Table 2:** The *in vitro* cytotoxicity for the studied complexes 1–8 and *cisplatin* against a panel of human cancer cell lines (HOS, G361, MCF7, A549, HeLa, 22Rv1, A2780, A2780R and Caco-2) and human non-cancerous cell line (Hep). Experiments included 24 h of drug exposure with no cell recovery in drug-free medium. Data are expressed as IC_50_±SD (μM) for all the used cancer cell lines and as IC_50_ (μM) for non-cancerous Hep cell line.

	HOS	G361	MCF7	A549	HeLa	22Rv1	A2780	A2780R	Caco-2	Hep
**1**	0.8±0.4***	2.9±0.6***	1.7±0.8***	12.3±1.1	7.0±0.6*	4.6±1.2***	3.5±0.7***	3.3±0.3	3.3±0.2	3.9
**2**	1.3±0.8***	2.3±1.0**	1.5±0.4***	6.4±1.4	4.8±0.4*	4.8±2.4***	3.7±0.4***	3.3±0.5	3.1±0.2	5.9
**3**	2.2±1.2***	3.1±0.2**	1.9±0.5***	8.8±2.2	4.7±0.8*	4.2±1.2***	2.8±0.3***	3.2±0.2	3.0±0.1	3.9
**4**	2.8±1.0***	1.6±0.7***	1.8±0.3***	9.8±1.3	6.2±0.7*	4.5±1.6***	2.3±1.1***	2.6±0.8	3.3±0.1	11.8
**5**	0.5±0.2***	3.2±0.2***	1.5±0.5***	4.7±0.3	3.8±0.3*	4.2±0.1***	3.1±0.1***	3.4±0.2	2.4±0.7	4.2
**6**	0.4±0.1***	3.2±0.2***	1.0±0.4***	4.3±0.9	3.8±0.2*	3.8±0.1***	3.2±0.2***	3.3±0.4	0.4±0.3	4.1
**7**	1.4±1.1***	3.4±0.3***	1.6±0.8***	7.3±1.6	5.4±1.2*	5.1±0.8***	3.4±0.2***	3.5±0.5	3.6±0.2	3.8
**8**	0.7±0.2***	1.7±1.3**	2.1±1.0***	3.5±0.2	3.8±0.1*	3.5±0.2***	1.7±0.3***	1.0±0.4	0.4±0.2	9.5
*Cisplatin*	18.9±1.7	5.3±0.7	17.9±3.5	>50.0	30.4±11.0	26.9±3.5	28.1±0.9	>50.0	>90.0	>75.0

The significant differences between the IC_50_ values for 1–8 and *cisplatin* are given as follows:

* *p* < 0.05,

** *p* < 0.01,

*** *p* < 0.005.

**Fig 3:**
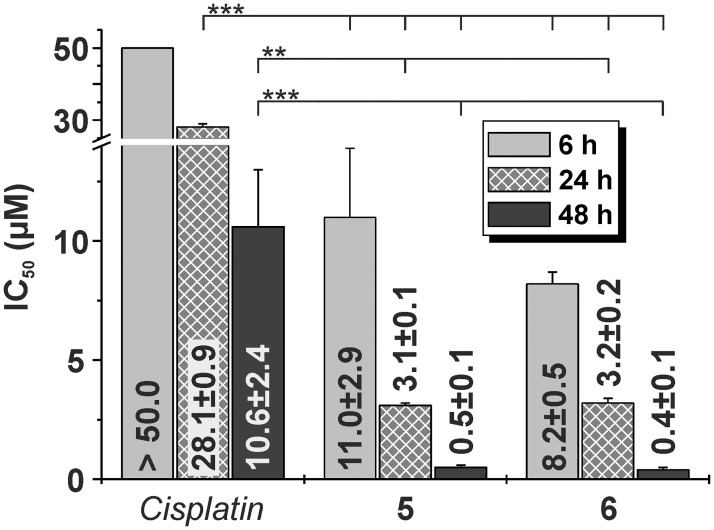
The results of time-dependent *in vitro* cytotoxicity experiments for the representative complexes 5 and 6, and *cisplatin*, as performed on the A2780 human ovarian carcinoma cell line with 6, 24 and 48 h exposure times. The significant difference between the IC_50_ values of complexes 5 and 6 and *cisplatin* is given as ** for *p* < 0.01 and *** for *p* < 0.005.

All the complexes **1**–**8** showed considerably higher activity than both reference drugs (*cisplatin* and *oxaliplatin*). Complexes **5**, **6** and **8** were identified as the most effective substances against all cell lines and were found to be up to ca 47-times more potent than *cisplatin* against HOS cell line. In the time-resolved experiments, the representative complexes **5** and **6** showed more rapid onset of cytotoxicity than *cisplatin* in the A2780 cells (Fig 3). To the best of our knowledge, there are only few examples of platinum(II) diiodido complexes containing two monodentate or one bidentate *N*-donor ligand/s which showed at least 5-fold higher activity than *cisplatin*. In particular, *cis*-[PtI_2_(*ip*am)_2_] was found to be up to ca 11-times more potent than *cisplatin* against A2780, T-47D human breast carcinoma and WiDr human colon carcinoma cell lines [9]. The complex *trans*-[PtI_2_(L_1_)_2_] exceeded activity of *cisplatin* against the HeLa cells approximately by the factor of 6 (L_1_ = dimethylamine) [10]. The effectivity of [PtI_2_(L_2_)] in the EA.hy 926 cancer cells was ca 6-times higher than that of *cisplatin*; L_2_ = isobutyl ester of (*S*,*S*)-1,3-propanediamine-*N*,*N`*-di-2-(cyclohexyl)propanoic acid; EA.hy 926 = derived by fusing human umbilical vein endothelial cells with A549 cells [11]. However, only the platinum(II) iodido complexes of the different types, such as mixed-ligand [13] or dinuclear [14] *N-*heterocyclic carbene complexes, showed *in vitro* anticancer activity comparable with **1**–**8**, but the mentioned substances were found to be toxic to the non-cancerous MRC-5 human fibroblasts (see below).

The high activity of complexes **1**–**8** against the Caco-2 cells, presumed to be intrinsic resistant to *cisplatin* [34], and A2780R *cisplatin*-resistant subline of ovarian cancer cell line indicated the ability of complexes **1**–**8** to overcome intrinsic, as well as acquired resistance of the cancer cells to *cisplatin*. The resistance factor (RF) values for the given complexes **1**–**8**, defined as the ratio IC_50_(A2780R)/IC_50_(A2780) ranged from 0.59 to 1.14 (S4 Table). These results are comparable with those obtained for the best performing platinum(II) iodido complexes published to date, such as diiodido complexes containing different 1,2-bis(aminomethyl)cyclohexane derivatives, which showed on the same pair of cell lines the resistance factors in the range 1.1–4.1 [35]. Moreover, the RF values obtained for complexes **1**–**8** are lower than for *picoplatin* (RF = 2.0 as observed in the A2780 and A2780R pair of ovarian carcinoma cell lines used in the present work as well) [36].

In comparison with the second reference drug *oxaliplatin* (IC_50_ > 25.0 μM), complexes **1**–**8** prevailed also in the cytotoxicity against the colon carcinoma Caco-2 cells (Table 2). Although a lot of platinum(II) iodido complexes have been studied for their activity in the colon carcinoma cells [6,9,10,13,14], not many of them were referenced to the activity of *oxaliplatin*, and even less platinum(II) iodido complexes exceeded its activity. The latter group is represented for example by the monofunctional ionic complex [PtI(mecyt)(mephen)]I (mecyt = 1-methylcytosine; mephen = 2,9-dimethyl-1,10-phenanthroline), which showed higher activity (IC_50_ = 0.2–0.5 μM) against HCT15, DLD-1, LoVo and *oxaliplatin*-resistant LoVo-OXP colon cancer cell lines than *oxaliplatin* (IC_50_ = 1.2–16.4 μM) [8]. For comparison, *in vitro* potency of complexes **1**–**8** against the colon carcinoma is higher than that of *picoplatin*, which showed lower activity (IC_50_ = 12.5 and 17.0 μM) than *oxaliplatin* (IC_50_ = 0.3 and 0.7 μM) in the HCT116 and HT29 colon carcinoma cells [36–37].

The studies of toxicity of complexes **1**–**8** against the cell culture of normal human hepatocytes (Hep) allowed us to assess the selectivity between the cancer and healthy cells. Although the toxicity of complexes **1**–**8** was higher than that of *cisplatin* (Table 2), the selectivity factor values, calculated as a ratio of IC_50_(Hep)/IC_50_(cancer cell line), showed the pharmacologically prospective selectivity for complexes **6** and **8** against HOS (ca 10, and 14, respectively) and Caco-2 (ca 10, and 24, respectively). Only a few of platinum(II) iodido complexes have been studied for their selective anticancer activity to date. Mixed-ligand complexes containing *N-*heterocyclic carbene ligands, such as *trans*-[Pt(ach)I_2_(L_4_)] (L_4_ = 1-benzyl-3-methylimidazol-2-ylide) or the above-mentioned [PtI_2_(L_2_)] complex and its analogues containing different esters of (*S*,*S*)-1,3-propanediamine-*N*,*N`*-di-2-(cyclohexyl)propanoic acid, showed comparable *in vitro* cytotoxicity in the cancerous and non-cancerous cells, indicating their low selectivity [11,13].

The above presented results of *in vitro* antiproliferative screening studies showed not only high activity and ability to overcome the resistance of some cancer cells to *cisplatin*, but also high potential of selected complexes (*i*.*e*. complexes **5**, **6** and **8**) for further testing on *in vivo* model of anticancer activity and mechanistic aspects of their interactions with biomolecules and biological systems.

### Hydrophobicity Studies (log*P* Determination) and Cellular Accumulation

The log*P* values for octanol/water system were determined only for the representative complexes **4** and **6** (due to their different *in vitro* cytotoxicity, see Table 2). The values of log*P* equalled 0.41 for complex **4** and –0.07 for complex **6** indicating that the prepared complexes are markedly more hydrophobic than *cisplatin* with log*P* = –2.19 [38].

As it is known and generally accepted for biologically potent transition metal complexes and reported also for several platinum(II) iodido complexes,^6,11,14,32^ substances with higher cellular accumulation usually show higher anticancer potency. For example, the highly antitumour active *cis*-diiodidoplatinum(II) complex containing acetone oxime showed markedly higher cellular accumulation than less potent dichlorido and dibromido analogues and *cisplatin* [39]. Herein reported complexes **4** and **6**, which were found to be markedly more hydrophobic and *in vitro* anticancer active against the A2780 cells than *cisplatin*, showed comparable (complex **6**; 598±97 pmol Pt/10^6^ A2780 cells), or slightly lower (complex **4**; 354±63 pmol Pt/10^6^ A2780 cells) cellular accumulation as compared with *cisplatin* (535±16 pmol Pt/10^6^ A2780 cells). For complexes **4** and **6**, the observed cellular accumulation rate (**4** < **6**) correlated with differences in their cytotoxicity (see Table 2).

### Cell Cycle Analysis

**Fig 4:**
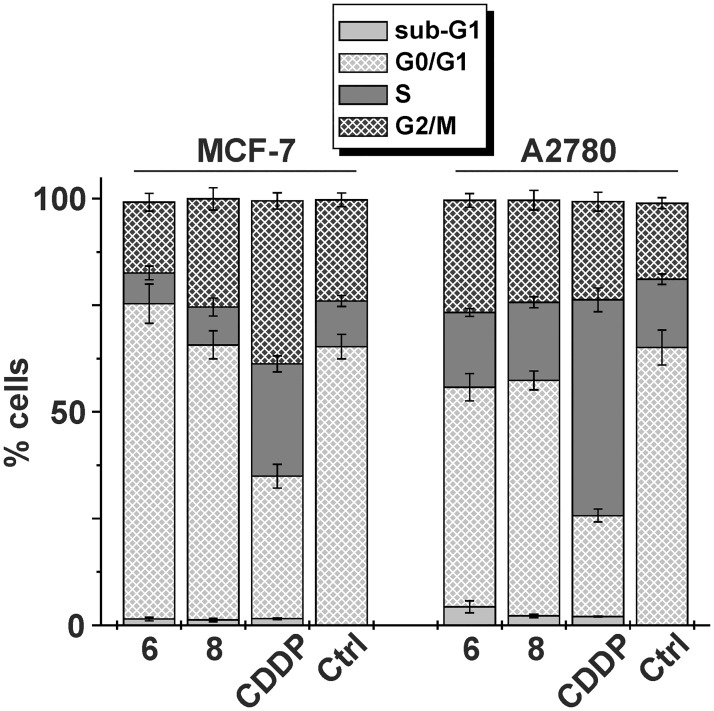
Cell populations (%) in sub-G1, G0/G1, S and G2/M cell cycle phases as observed on human cancerous MCF7 (left) and A2780 (right) cells after 24 h exposure to complexes 6 and 8 (and *cisplatin* for comparative purposes) at IC_50_ concentrations. Cells were stained with PI/RNase. The data are given as arithmetic mean±SD from three independent experiments conducted on cells from three consecutive passages.

Platinum-based chemotherapeutics [40] as well as other anticancer drugs [41] affect the processes involved in the progression of division of the treated cancer cells, resulting in perturbation of the normal distribution of cell populations in the different phases of the cell cycle. In this work, the cell cycle modifications were analyzed for the representative complexes **6** and **8** (and *cisplatin* for comparative purposes) in MCF7 and A2780 human cancer cells (Fig 4 and S5 Table).

Both the studied complexes showed similar effects on the cell cycle in both cell lines used (Fig 4). Surprisingly, the distribution of the cells treated with complexes **6** and **8** (applied at the IC_50_ concentrations) in different cell cycle phase populations was comparable with the untreated (control) cells and markedly different from *cisplatin* [6,42]. In comparison with the untreated MCF-7 cells, the application of complex **6** induced a slight increase of their G0/G1 populations. Contrary, the G0/G1 populations of the A2780 cells were lower after the treatment with complexes **6** and **8** than for the control A2780 cells, while the G2/M populations of these cells treated by complexes **6** and **8** increased in comparison with control. Treatment by complexes **6** and **8** led to the cell cycle perturbations different from recently reported *cis*- and *trans*-platinum(II) diiodido complexes [10] as well as from mixed-ligand platinum(II) dichlorido complexes containing 7-azaindole-based *N*-donor ligand [6], whose application resulted in higher S cell cycle phase populations. Only the low portion of populations (1.2–4.3%) corresponding to apoptotic and necrotic cells were identified in the sub-G1 region after 24 h exposure to complexes **6** and **8** (Fig 4), which is considerably different than recently reported for dichlorido analogues (sub-G1 populations of ca 30%) of the herein studied platinum(II) diiodido complexes [43]. *Cisplatin*, on the other hand, showed a typical increase of the S cell cycle phase populations in both the cancer cell lines [6,10,42,43], resulting in higher S and lower G0/G1 populations than observed for complexes **6** and **8** (and control cells) on both MCF-7 and A2780 cancer cells (Fig 4). Taken together, application of complexes **6** and **8** led to the cell cycle perturbations indirectly indicating different mechanism of action from *cisplatin*.

### Interactions with dsDNA

**Fig 5:**
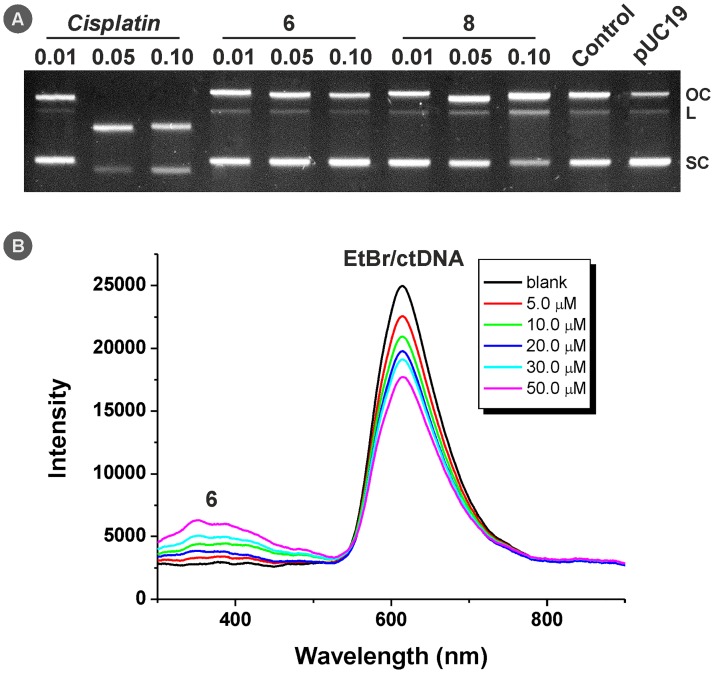
(A) The results of gel electrophoretic studies of interaction of complexes 6 and 8 (and cisplatin for comparative purposes) with pUC19 plasmid dsDNA. (B) The results of fluorescence quenching experiments on the EtBr/ctDNA system for complex 6. Experiment was performed on plasmid dsDNA in the used medium (labeled as Control) and in water (labeled as pUC19).

The studies were performed by means of the mobility shift and/or fragmentation tests using the gel electrophoresis of the reaction intermediates formed with pUC19 plasmid dsDNA, and the fluorescence quenching experiments of the EtBr/ctDNA system. The representative results for complexes **6** and **8** are given in Fig 5.

The results of gel electrophoresis of the reaction intermediates of pUC19 plasmid DNA with complexes **6**, **8** and *cisplatin* showed that complexes **6** and **8** did not significantly change the mobility of the supercoiled (SC) form or other forms (i.e. open circular (OC) form or linearized (L) form) of pUC19 in contrast to *cisplatin*, which probably due to intramolecular cross-linking helped to increase the electrophoretic mobility of former SC form of pUC19 dsDNA [44]. The same effect was observed for OC form of pUC19 plasmid DNA, in which the formation of intramolecular cross-links led to increased mobility. These observations suggest on the lower reactivity in the means of coordination (covalent modifications) or different type of interactions of complexes **6** and **8** with dsDNA in comparison with *cisplatin* (Fig 5).

On the other hand, complex **8** visibly increased the amount of single strand-damaged OC form, as well as double strand-damaged and cleaved linear (L) form of pUC19 DNA in concentration-dependent manner (Fig 5 and S6 Table). These results may bring an indirect proof of different mechanism of action of the studied complexes from *cisplatin*, in terms of different types of interactions with dsDNA, for example by its cleavage via various possible mechanisms (e.g. photo-activation, radical cleavage, etc.) [45–46].

The results of the fluorescence quenching experiments performed for complexes **6** and **8** (and *cisplatin* for comparative purposes) on the EtBr/ctDNA system showed the ability of these compounds to decrease the fluorescence intensity of the EtBr/ctDNA supramolecular complex with the increasing concentration of the quencher. This effect is known for compounds covalently binding to ctDNA (including *cisplatin*) [47], as well as for the substances intercalating into the structure of DNA (including free 7-azaindoles) [48–49]. The Stern-Volmer and apparent binding constants for complexes **6** (*K*_SV_ = 7.4 × 10^3^ M^–1^, *K*_app_ = 7.8 × 10^4^ M^–1^) and **8** (*K*_SV_ = 1.4 × 10^4^ M^–1^, *K*_app_ = 1.2 × 10^5^ M^–1^), were higher than those for *cisplatin* (*K*_SV_ = 4.7 × 10^3^ M^–1^, *K*_app_ = 4.8 × 10^4^ M^–1^) and showed on moderate binding affinity of complexes **6** and **8** to the ctDNA (Fig 5).

### *In Vivo* Antitumour Activity

**Fig 6:**
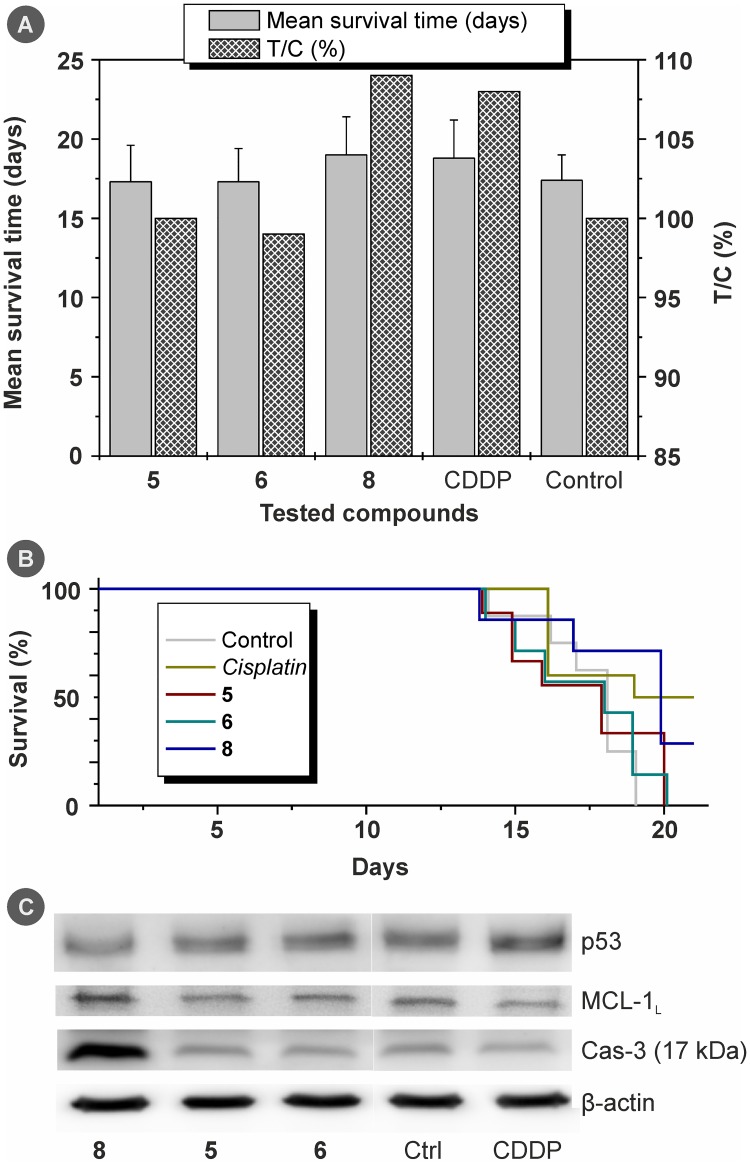
(A) *In vivo* antitumour activity for complexes 5, 6 and 8 (and *cisplatin* for comparative purposes), as investigated on L1210 leukaemia-bearing mice, and given as mean survival times ± SD (days) and T/C (%) values; (B) Kaplan-Meier plots showing the percentage of survival of animals in the individual groups (days after tumour implantation); (C) the results for complexes 5, 6 and 8 on the p53, MCL-1_L_ (37 kDa) and Cas-3 (17 kDa) expression as obtained by Western blot analyses in the samples of collected tumour tissues.

The selected representative complexes **5**, **6** and **8** (chosen for *in vivo* studies based on their high *in vitro* cytotoxicity and relatively good solubility) were tested together with *cisplatin* for *in vivo* antitumour activity using the implantation model of L1210 lymphocytic leukaemia in mice. The maximum tolerated dose of *cisplatin* in our experiments was 2 mg/kg and the respective doses of complexes **5**, **6** and **8** were recalculated to be equimolar (*i*.*e*. contain the same amount of Pt) to *cisplatin*. The mean survival times for the treated groups (T) were compared with the intact control group (C) and expressed as a relative percentage of the survival time extension T/C (%). These findings, together with Kaplan-Meier survival curves for all groups of animals and the results of *ex-vivo* Western blot analyses are shown in Fig 6.

The tested complexes **5**, **6** and **8** were well-tolerated and no signs of their toxicity (e.g. weight loss) were observed during experiment. The applied dose of complexes **5**, **6** and **8** showed different effects on the treated mice. In particular, only complex **8** enhanced the survival time (19.0±2.4 days) as compared with the control group (17.4±1.6 days), while survival time of mice treated by complex **5** (17.3±2.3 days) and complex **6** (17.3±2.1 days) was found to be comparable to control group (Fig 6). *In vivo* antitumour effect of complex **8** was comparable with *cisplatin* (18.8±2.4 days). The obtained survival time results expressed as the percentage of mean survival time (T/C; %) were 100%, 99% and 109% for complexes **5**, **6** and **8**, respectively, and 108% for *cisplatin*. In comparison with the dichlorido analogues of herein tested diiodido complexes, complex **8** exceeded significantly the average T/C values previously reported for the mentioned dichlorido complexes applied at 2 mg/kg dose, lying in the range between 97.1 and 100.0% [17]. The similar results, as for the survival times, were also reported for *cis*-[PtCl_2_{*E*-N(H) = C(OMe)CH_2_Ph}_2_] complex applied at 2.5 mg/kg dosage in similar experimental model [50].

### Histological and Protein Expression *Ex Vivo* Analysis

In order to better understand the cellular effects of the tested complexes on the cancerous as well as the healthy tissues, different tissues (diffuse and well-circumcised tumours, spleen, and renal tissue with intact perirenal fat) were collected from the sacrificed mice post mortem and studied by means of optical microscopy (after standard hematoxylin and eosin staining) and electron microscopy. The infiltration rate of normal tissues by polymorphonuclear (PMN) cells was used as a sign of possible toxic effects of applied compounds and the average amount of cell deaths (mostly of necrosis origin) has been evaluated in tumour tissue samples. The semi-quantitative evaluations of these indicators are summarized in Table 3.

**Table 3:** The semi-quantitative evaluation* of PMN cells infiltration of spleen and renal tissues and the average number of cells undergoing the cell death (mostly necrosis) in the tumour tissues obtained from the animals of individual groups treated with the selected compounds and untreated control involved in testing of *in vivo* anticancer activity. The relative scale from 0 to 4 was used, where 0 = without necrosis, 1 = up to 25%, 2 = up to 50%, 3 = up to 75% and 4 = up to 100% of the areas in the view-field.

Group	Infiltration of spleen	Infiltration of renal tissues	Areas of necrosis in tumour tissues
**5**	3	1	2
**6**	2	1	1–2
**8**	1–2	0–1	3
*Cisplatin*	1	1	3
Control	0	0	1–2

**Fig 7:**
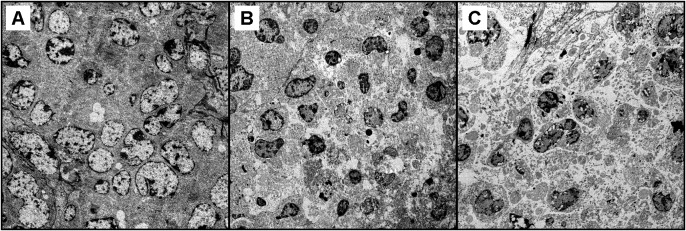
Electron micrograph image of (A) tumour cells in the control group sample showing no significant changes in chromatin structure and/or mitochondria shape; (B) a necrotic part of the tissue from the *cisplatin* treated group showing the late stages of cell death (mostly necrosis) with specific vacuolization in the cells nuclei and destruction of the cell membranes accompanied by the mitochondria swelling; (C) a focal very late stage of necrosis in the tumour tissue from the complex 6 treated group with almost complete destruction of the cells, advanced vacuolization and cell debris autophagy including the remnants of cell organelles.

The results of light microscopy observations clearly supported the results of life-span evaluation, and complex **8** was identified as the most effective in the induction of cell deaths in tumour tissues and at the same time only slightly toxic towards the normal tissues (spleen and renal tissues). Its effects were comparable with *cisplatin*. The representative micrograph image, depicting the amount and distribution of necrotic processes in the tumour tissues, has been included in S7 Fig. To evaluate the sub-cellular processes in the tumour tissues, the tissue samples from the control group, *cisplatin* treated group and complex **6** treated group were processed for electron microscopy observations. The representative micrograph images depict the usual appearance of the untreated tumour cells (Fig 7A), the late stages of necrotic processes in the *cisplatin* treated tissue (Fig 7B) and the focal very late stages of complete tumour tissue destruction by the necrosis (and partly apoptosis and/or necroptosis) induced by the application of complex **6** (Fig 7C).

The anti-cancer effect of the tested complexes was also evaluated on the molecular level using the collected tumour tissue samples from the individual groups involved in the *in vivo* testing as described above. The expression of tumour suppressor p53, anti-apoptotic protein MCL-1_L_ (37 kDa) and the cleavage of caspase 3 (Cas-3) into active pro-apoptotic form p17 was detected in the tumour tissue (Fig 6C). Lowering of levels and/or dysfunction of p53 is one of the best known mechanisms of the cancerogenesis and it is also connected with chemotherapy resistance [51]. Our previously published work showed that *cisplatin* was able to increase the p53 level in the L1210 cell-induced murine cancer model, but *cis*-[PtCl_2_(*n*aza)_2_] complexes, that are previously reported dichlorido-analogues of the herein presented complexes, had lower effect or even decreased its level [17]. Similarly, complex **8** attenuated the amount of p53 but complexes **5** and **6** did not affect the p53 level at all. The p53 protein could trigger the mitochondrial (intrinsic) apoptosis via the activation of Noxa and subsequently inhibition of MCL-1_L_ [52]. Complexes **5** and **6** had only negligible effect on the quantity of MCL-1_L_, corresponding to their inability to modulate p53. However, complex **8** considerably increased the MCL-1_L_ level, probably due to decreased amount of p53. Interestingly, although complex **8** attenuated p53 expression and augmented MCL-1_L_, it also dramatically increased the level of active form of Cas-3. Perhaps, this could be a result of the activation of p53-independent pathway, as it has been previously demonstrated for different platinum(II) complex [53]. Activation of Cas-3 has not been observed for other tested compounds (**5**, **6** and *cisplatin*). Higher degree of Cas-3 activation by complex **8** corresponds with its ability to prolong life-span of mice with induced tumourigenesis.

## Conclusions

Eight platinum(II) diiodido complexes containing 7-azaindole-based *N*-donor ligands showed a wide-range *in vitro* antitumour activity against nine human cancer cell lines, including Caco-2 colon carcinoma cells and A2780R variant of ovarian carcinoma cells with acquired resistance to conventional *cisplatin*. Compounds **6** and **8** with the highest *in vitro* potency exhibited pronounced selectivity towards HOS human osteosarcoma and Caco-2 cancer cells over normal human hepatocytes, with the selectivity factor up to ca 24. Enhancement of the survival time of L1210 lymphocytic leukaemia-bearing mice treated with complex **8** indicated this substance as a possible candidate for further preclinical studies.

On the molecular level, the treatment with complex **8** led to the decrease of tumour suppressor p53 amount and increase of the levels of anti-apoptotic protein MCL-1_L_ (37 kDa) and active pro-apoptotic form of caspase 3, possibly showing on the p53-independent cell death pathway different from *cisplatin*. The microscopic observations of necrosis as the main cell death mechanism and the results of A2780 and MCF-7 cell cycle perturbation studies or gel electrophoretic studies on plasmid DNA were also suggestive for different mechanism of action as compared to clinically used platinum-based drug *cisplatin*.

## Supporting Information

S1 Table Crystal data and structure refinements for *cis*-[PtI_2_(*2Me4Cl*aza)_2_]∙DMF (8∙DMF). (PDF)

S1 Text ^1^H–^1^H gs-COSY and ^1^H–^13^C gs-HMQC two dimensional NMR spectra of the representative complexes 3, 7 and 8. The complexes were dissolved in DMF-*d*_*7*_.
(DOCX)

S1 File Crystallographic information file containing the structural data for complex 8∙DMF. (CIF)

S2 Table The ^1^H and ^13^C NMR coordination shifts (calculated as Δδ = δ_complex_− δ_ligand_; ppm) of the prepared complexes. (PDF)

S2 Text ESI-MS data for complexes 1–8. (PDF)

S1 Fig ESI–mass spectrum of the representative complex *cis*-[PtI_2_(*5Br*aza)_2_] (7). The complex was dissolved in methanol and an assignment of the observed peaks and details of experimental (black) and simulated (grey) isotopic distribution of the {[PtI_2_(*5Br*aza)_2_]–H}^−^(green) and {[PtI_2_(*5Br*aza)]–H}^−^(orange) species is included.
(TIF)

S2 Fig ^1^H NMR spectra recorded at different time points at 293 K on 1 mM 20% DMF-*d*_*7*_/80% D_2_O solution of *cis*-[PtI_2_(*4Br*aza)_2_] (6), given together with ^1^H NMR spectrum of free *4Br*aza dissolved in the same mixture of solvents. The signals of complex 6 are marked with circles and the signals of the hydrolysed form of complex 6 are marked with squares.
(TIF)

S3 Fig ^1^H NMR spectra recorded at different time points at 293 K on 1 mM 20% DMF-*d*_*7*_/80% D_2_O solution of *cis*-[PtI_2_(*4Br*aza)_2_] (6) mixed with reduced glutathione (GSH). The signals of complex **6** are marked with red circles, the signals of the hydrolysed form of complex **6** are marked with green squares and blue circles mark the signals of GSH.
(TIF)

S4 Fig ^1^H NMR spectra recorded at different time points at 293 K on 1 mM 20% DMF-*d*_*7*_/80% D_2_O solution of *cis*-[PtI_2_(*4Br*aza)_2_] (6) or *cisplatin* (orange rectangle), both mixed with GMP. The signals of complex 6 are marked with red circles, the signals of the hydrolysed form of complex 6 are marked with green squares, blue circles mark the signals of GMP and yellow squares assign Pt–GMP adduct.
(TIF)

S5 Fig Hydrogen bonding (drawn in light blue) within the crystal structure of *cis*-[PtI_2_(*2Me4Cl*aza)_2_]∙DMF (8∙DMF). The hydrogen atoms not involved in the depicted hydrogen bonds were omitted for clarity.
(TIF)

S3 Table The parameters of the non-covalent contacts in the crystal structure of *cis*-[PtI_2_(*2Me4Cl*aza)_2_]∙DMF (8∙DMF). (PDF)

S6 Fig Part of the crystal structure of *cis*-[PtI_2_(*2Me4Cl*aza)_2_]∙DMF (8∙DMF) showing the formation of supramolecular structure through the selected C2A⋯Cl1, C3A⋯Cl1 and C5A–H⋯I1 non-covalent contacts (orange dashed lines). The hydrogen atoms not involved in the depicted non-covalent contacts and DMF molecule of crystallization were omitted for clarity.
(TIF)

S4 Table The resistance factors, defined as the ratio between *in vitro* cytotoxicity (IC_50_) against resistant and sensitive variants of the A2780 ovarian cancer cell line (IC_50_-A2780R/IC_50_-A2780), calculated for complexes 1–8 and *cisplatin*. (PDF)

S5 Table The sub-G1, G0/G1, S and G2/M populations (%) detected at MCF7 and A2780 human cancer cell lines treated with complexes 6 and 8, and *cisplatin* for comparative purposes. The experimental conditions were as follows 24 h exposure time; IC_50_ concentrations applied and stained with PI/RNase. The data are given as arithmetic mean±SD from three independent experiments.
(PDF)

S7 Fig A representative light micrograph image depicting the tumour tissue (lymphoma) with focal necrosis (N). The tissue sample was stained by standard hematoxylin and eosin staining and photographed at 100× magnification.
(JPG)

## References

[1] Wheate NJ, Walker S, Craig GE, Oun R. The status of platinum anticancer drugs in the clinic and in clinical trials. Dalton Trans. 2010; 39: 8113–8127. 10.1039/c0dt00292e 20593091

[2] Kelland L. The resurgence of platinum-based cancer chemotherapy. Nature Rev Cancer. 2007; 7: 573–584. 17625587 10.1038/nrc2167

[3] Cleare MJ, Hoeschele JD. Studies on the antitumour activity of group VIII transition Metal complexes. Part I. Platinum(II) complexes. Bioinorg Chem. 1973; 2: 187–210.

[4] Aggarwal SK, Broomhead JA, Fairlie DP, Whitehouse MW. Platinum drugs: Combined anti-lymphoproliferative and nephrotoxicity assay in rats. Cancer Chemother Pharmacol. 1980; 4: 249–258. 7438327 10.1007/BF00255269

[5] Arandjelovic S, Tesic Z, Juranic Z, Radulovic S, Vrvic M, Potkonjak B, et al Antiproliferative activity of some *cis*-/*trans*-platinum(II) complexes on HeLa cells. J Exp Clin Cancer Res. 2002; 21: 519–526. 12636098

[6] Marzo T, Pillozzi S, Hrabina O, Kasparkova J, Brabec V, Arcangeli A, et al *cis*-PtI_2_(NH_3_)_2_: a reappraisal. Dalton Trans. 2015; 44: 14896–14905. 10.1039/c5dt01196e 26226326

[7] Margiotta N, Natile G, Capitelli F, Fanizzi FP, Boccarelli A, De Rinaldis P, et al Sterically hindered complexes of platinum(II) with planar heterocyclic nitrogen donors. A novel complex with 1-methyl-cytosine has a spectrum of activity different from cisplatin and is able of overcoming acquired cisplatin resistance. J Inorg Biochem. 2006; 100: 1849–1857. 10.1016/j.jinorgbio.2006.07.010 16959321

[8] Margiotta N, Savino S, Gandin V, Marzano C, Natile G. Monofunctional platinum(II) complexes with potent tumour cell growth inhibitory activity: the effect of a hydrogen-bond donor/acceptor N-heterocyclic ligand. ChemMedChem. 2014; 9: 1161–1168. 10.1002/cmdc.201402028 24799414

[9] Messori L, Casini A, Gabbiani C, Michelucci E, Cubo L, Rios-Luci C, et al Cytotoxic profile and peculiar reactivity with biomolecules of a novel “rule-breaker” iodidoplatinum(II) complex. ACS Med Chem Lett. 2010; 1: 381–385. 10.1021/ml100081e 24900222 PMC4007831

[10] Messori L, Cubo L, Gabbiani C, Alvarez-Valdes A, Michelucci E, Pieraccini G, et al Reactivity and biological properties of a series of cytotoxic PtI_2_(amine)_2_ complexes, either *cis* or *trans* configured. Inorg Chem. 2012; 51: 1717–1726. 10.1021/ic202036c 22225466

[11] Savic A, Filipovic L, Arandjelovic S, Dojcinovic B, Radulovic S, Sabo TJ, et al Synthesis, characterization and cytotoxic activity of novel platinum(II) iodido complexes. Eur J Med Chem. 2014; 82: 372–384. 10.1016/j.ejmech.2014.05.060 24927057

[12] Skander M, Retailleau P, Bourrie B, Schio L, Mailliet P, Marinetti A. N-heterocyclic carbene-amine Pt(II) complexes, a new chemical space for the development of platinum-based anticancer drugs. J Med Chem. 2010; 53: 2146–2154. 10.1021/jm901693m 20148592

[13] Chardon E, Dahm G, Guichard G, Bellemin-Laponnaz S. Derivatization of preformed platinum N-heterocyclic carbene complexes with amino acid and peptide ligands and cytotoxic activities toward human cancer cells. Organometallics. 2012; 31: 7618–7621.

[14] Chtchigrovsky M, Eloy L, Jullien H, Saker L, Segal-Bendirdjian E, Poupon J, et al Antitumour *trans*-N-heterocyclic carbene-amine-Pt(II) complexes: synthesis of dinuclear species and exploratory investigations of DNA binding and cytotoxicity mechanisms. J Med Chem. 2013; 56: 2074–2086. 10.1021/jm301780s 23421599

[15] Messori L, Marzo T, Gabbiani C, Valdes AA, Quiroga AG, Merlino A. Peculiar features in the crystal structure of the adduct formed between *cis*-PtI_2_(NH_3_)_2_ and hen egg white lysozyme. Inorg Chem. 2013; 52: 13827–13829. 10.1021/ic402611m 24256441

[16] Štarha P, Trávníček Z, Popa A, Popa I, Muchová T, Brabec V. How to modify 7-azaindole to form cytotoxic Pt(II) complexes: highly in vitro anticancer effective cisplatin derivatives involving halogeno-substituted 7-azaindole. J Inorg Biochem. 2012; 115: 57–63. 10.1016/j.jinorgbio.2012.05.006 22922312

[17] Štarha P, Hošek J, Vančo J, Dvořák Z, Suchý P, Popa I, et al Pharmacological and molecular effects of platinum(II) complexes involving 7-azaindole derivatives. PLoS ONE. 2014; 9: e90341 10.1371/journal.pone.0090341 24603594 PMC3948342

[18] Štarha P, Dvořák Z, Trávníček Z. Highly and broad-spectrum in vitro antitumour active *cis*-dichloridoplatinum(II) complexes with 7-azaindoles. PLoS ONE. 2015; 10: e0136338 10.1371/journal.pone.0136338 26309251 PMC4550364

[19] New EJ, Roche C, Madawala R, Zhang JZ, Hambley TW. Fluorescent analogues of quinoline reveal amine ligand loss from *cis* and *trans* platinum(II) complexes in cancer cells. J Inorg Biochem. 2009; 103: 1120–1125. 10.1016/j.jinorgbio.2009.05.005 19564043

[20] Pracharova J, Saltarella T, Radosova Muchova T, Scintilla S, Novohradsky V, Novakova O, et al Novel antitumour cisplatin and transplatin derivatives containing 1-methyl-7-azaindole: synthesis, characterization, and cellular responses. J Med Chem. 2015; 58: 847–859. 10.1021/jm501420k 25496325

[21] Štarha P, Trávníček Z, Dvořák Z, Radošová-Muchová T, Prachařová J, Vančo J, et al Potentiating effect of UVA irradiation on anticancer activity of carboplatin derivatives involving 7-azaindoles. PLoS ONE. 2015; 10: e0123595 10.1371/journal.pone.0123595 25875850 PMC4398499

[22] Oxford Diffraction, CrysAlis RED and CrysAlis CCD Software (Ver. 1.171.33.52), Oxford Diffraction Ltd., Abingdon, Oxfordshire, UK.

[23] Sheldrick GM. Crystal structure refinement with SHELXL. Acta Crystallogr. C. 2015; 71: 3–8. 10.1107/S2053229614024218 PMC4294323 25567568

[24] Brandenburg K, Diamond Version 4.0.3., Crystal Impact GbR, Bonn, Germany, 2015.

[25] Macrae CF, Bruno IJ, Chisholm JA, Edgington PR, McCabe P, Pidcock E, et al Mercury CSD 2.0—new features for the visualization and investigation of crystal structures. J Appl Crystallogr. 2008; 41: 466–470.

[26] Hissler M, Connick WB, Geiger DK, McGarrah JE, Lipa D, Lachicotte RJ, et al Platinum diimine bis(acetylide) complexes: synthesis, characterization, and luminescence properties. Inorg Chem. 2000; 39: 447–457. 11229561 10.1021/ic991250n

[27] Vardevanyan PO, Antonyan AP, Parsadanyan MA, Davtyan HG, Karapetyan AT. The binding of ethidium bromide with DNA: interaction with single- and double-stranded structures. Exp Mol Med. 2003; 35: 527–533. 10.1038/emm.2003.68 14749530

[28] Dhara SC. A rapid method for the synthesis of *cis*-[Pt(NH_3_)_2_Cl_2_]. Indian J Chem. 1970; 8: 193–194.

[29] Gulyaeva N, Zaslavsky A, Lechner P, Chlenov M, Chait A, Zaslavsky B. Relative hydrophobicity and lipophilicity of β-blockers and related compounds as measured by aqueous two-phase partitioning, octanol-buffer partitioning, and HPLC. Eur J Pharm Sci. 2002; 17: 81–93. 12356423 10.1016/s0928-0987(02)00146-x

[30] Tetko IV, Varbanov HP, Galanski M, Talmaciu M, Platts JA, Raverag M, et al Prediction of logP for Pt(II) and Pt(IV) complexes: comparison of statistical and quantum-chemistry based approaches. J Inorg Biochem. 2016; 156: 1–13. 10.1016/j.jinorgbio.2015.12.006 26717258

[31] Štarha P, Marek J, Trávníček Z. Cisplatin and oxaliplatin derivatives involving 7-azaindole: structural characterisations. Polyhedron. 2012; 33: 404–409.

[32] Tessier C, Rochon FD. Multinuclear NMR study and crystal structures of complexes of the types *cis*- and *trans*-Pt(Ypy)_2_X_2_, where Ypy = pyridine derivative and X = Cl and I. Inorg Chim. Acta. 1999; 295: 25–38.

[33] Sakai K, Yokoyama Y, Masaoka S. *cis*-Dichlorobis(4-methylpyridine-κN)platinum(II). Acta Cryst. 2007; E63: m97–m99.

[34] Shi Y, Tang B, Yu PW, Tang B, Hao YX, Lei X, et al Autophagy protects against oxaliplatin-induced cell death via ER stress and ROS in Caco-2 cells. PLoS ONE. 2012; 7: e51076 10.1371/journal.pone.0051076 23226467 PMC3511352

[35] de Mier-Vinue J, Lorenzo J, Montana AM, Moreno V, Aviles FX. Synthesis, DNA interaction and cytotoxicity studies of *cis*-{[1,2-bis(aminomethyl)cyclohexane]dihalo}platinum(II) complexes. J Inorg Biochem. 2008; 102: 973–987. 10.1016/j.jinorgbio.2007.12.026 18267344

[36] Holford J, Sharp SY, Murrer BA, Abrams M, Kelland LR. In vitro circumvention of cisplatin resistance by the novel sterically hindered platinum complex AMD473. Br J Cancer. 1998; 77: 366–373. 9472630 10.1038/bjc.1998.59 PMC2151285

[37] Sharp SY, O’Neill CF, Rogers PM, Boxall FE; Kelland LR. Retention of activity by the new generation platinum agent AMD0473 in four human tumour cell lines possessing acquired resistance to oxaliplatin. Eur J Cancer. 2002; 38: 2309–2315. 12441268 10.1016/s0959-8049(02)00244-7

[38] Ossipov K, Scaffidi-Domianello YY, Seregina IF, Galanski M, Keppler BK, Timerbaev AR, et al Inductively coupled plasma mass spectrometry for metallodrug development: albumin binding and serum distribution of cytotoxic *cis*- and *trans*-isomeric platinum(II) complexes. J Inorg Biochem. 2014; 137: 40–45. 10.1016/j.jinorgbio.2014.04.008 24803025

[39] Bartel C, Bytzek AK, Scaffidi-Domianello YY, Grabmann G, Jakupec MA, Hartinger CG, et al Cellular accumulation and DNA interaction studies of cytotoxic *trans*-platinum anticancer compounds. J Biol Inorg Chem. 2012; 17: 465–474. 10.1007/s00775-011-0869-5 22227950

[40] Ormerod M, O’Neill C, Robertson D, Kelland L, Harrap K. *cis*-Diamminedichloroplatinum(II)-induced cell death through apoptosis in sensitive and resistant human ovarian carcinoma cell lines. Cancer Chemother Pharmacol. 1996; 37: 463–471. 10.1007/s002800050413 8599870

[41] MacKeigan JP, Collins TS, Ting JPY. MEK inhibition enhances paclitaxel-induced tumour apoptosis. J Biol Chem. 2000; 275: 38953–38956. 10.1074/jbc.C000684200 11038347

[42] Wu S, Wang X, He Y, Zhu Z, Zhu C, Guo Z. A monofunctional trinuclear platinum complex with steric hindrance demonstrates strong cytotoxicity against tumour cells. J Inorg Biochem. 2014; 139: 77–84. 10.1016/j.jinorgbio.2014.06.006 24991692

[43] Muchová T, Prachařová J, Štarha P, Olivová R, Vrána O, Benešová B, et al Insight into the toxic effects of *cis*-dichloridoplatinum(II) complexes containing 7-azaindole halogeno derivatives in tumour cells. J Biol Inorg Chem. 2013; 18: 579–589. 10.1007/s00775-013-1003-7 23674329

[44] Onoa G Bibiana, Cervantes G, Moreno V, Prieto M José. Study of the interaction of DNA with cisplatin and other Pd(II) and Pt(II) complexes by atomic force microscopy. Nucleic Acids Res. 1998; 26: 1473–1480. 9490794 10.1093/nar/26.6.1473 PMC147433

[45] Berners-Price SJ. Activating platinum anticancer complexes with visible light. Angew. Chem. Int. Ed. 2011; 50: 804–805. 10.1002/anie.201004552 21246674

[46] Roy S, Maheswari PU, Lutz M, Spek AL, den Dulk H, Barends S, et al DNA cleavage and antitumour activity of platinum(II) and copper(II) compounds derived from 4-methyl-2-N-(2-pyridylmethyl)aminophenol: spectroscopic, electrochemical and biological investigation. Dalton Trans. 2009; 48: 10846–10860. 10.1039/b911542k 20023915

[47] Butour JL, Macquet JP. Differentiation of DNA-platinum complexes by fluorescence. The use of an intercalating dye as a probe. Eur J Biochem. 1977; 78: 455–463. 913408 10.1111/j.1432-1033.1977.tb11758.x

[48] Pasternack RF, Caccam M, Keogh B, Stephenson TA, Williams AP, Gibbst EJ. Long-range fluorescence quenching of ethidium ion by cationic porphyrins in the presence of DNA. J Am Chem Soc. 1991; 113: 6835–6840.

[49] Wanga K, Stringfellow S, Dong S, Jiao Y, Yu H. Synthesis and fluorescence study of 7-azaindole in DNA oligonucleotides replacing a purine base. Spectrochim. Acta A. 2002; 58: 2595–2603. 10.1016/s1386-1425(02)00004-5 PMC3764495 12396042

[50] Sbovata S Mazzega, Bettio F, Mozzon M, Bertani R, Venzo A, Benetollo F, et al Cisplatinum and transplatinum complexes with benzyliminoether ligands; synthesis, characterization, structure-activity relationships, and in vitro and in vivo antitumour efficacy. J Med Chem. 2007; 50: 4775–4784. 10.1021/jm070426p 17713897

[51] Siddik ZH. Cisplatin: mode of cytotoxic action and molecular basis of resistance. Oncogene. 2003; 22: 7265–7279. 10.1038/sj.onc.1206933 14576837

[52] Ploner C, Kofler R, Villunger A. Noxa: at the tip of the balance between life and death. Oncogene. 2009; 27: 84–92. 10.1038/onc.2009.46 PMC3272398 19641509

[53] Suntharalingam K, Wilson JJ, Lin W, Lippard SJ. A dual-targeting, p53-independent, apoptosis-inducing platinum(II) anticancer complex, [Pt(BDI(QQ))]Cl. Metallomics. 2014; 6: 437–443. 10.1039/c3mt00364g 24514456 PMC4082332

