# Supplementary material for: Platinum(II) Iodido Complexes of 7-Azaindoles with Significant Antiproliferative Effects: An Old Story Revisited with Unexpected Outcomes
Source: PLoS One. 2016 Dec 1;11(12):e0165062. doi: 10.1371/journal.pone.0165062 (PMC5131915; doi:10.1371/journal.pone.0165062)
Supplement: S1 Table — (PDF) [file pone.0165062.s001.pdf]

S1 Table. Crystal data and structure refinements for *cis*-[PtI_2(*2Me4Cl*aza)$_2$]·DMF (8·DMF).

Empirical formula	$C_{19}H_{21}N_5Cl_2I_2OPt$
Formula weight	855.20
Temperature (K)	120(2)
Wavelength (Å)	0.71073
Crystal system, space group	Triclinic, P-1
Unit cell dimensions	
a (Å)	11.0784(2)
b (Å)	11.0789(2)
c (Å)	11.6320(2)
α (°)	76.6972(17)
β (°)	65.0446(18)
γ (°)	71.7722(16)
V (Å^3)	1221.42(4)
Z, D_{calc} (g cm^{-3})	2, 2.325
Absorption coefficient (mm^{-1})	8.511
F (000)	792
θ range for data collection (°)	$2.905 \leq 2\theta \leq 24.998$
Reflections collected/unique (R_{int})	11162/4287 (0.0172)
Final R indices [I>2σ(I)]	R_1 = 0.0248, wR_2 = 0.0631
R indices (all data)	R_1 = 0.0268, wR_2 = 0.0639
Goodnes-of-fit	1.058
Largest peak and hole (e Å^{-3})	2.250, –0.868
