# Supplementary material for: Platinum(II) Iodido Complexes of 7-Azaindoles with Significant Antiproliferative Effects: An Old Story Revisited with Unexpected Outcomes
Source: PLoS One. 2016 Dec 1;11(12):e0165062. doi: 10.1371/journal.pone.0165062 (PMC5131915; doi:10.1371/journal.pone.0165062)
Supplement: S1 Text — The complexes were dissolved in DMF-d7. (DOCX) [file pone.0165062.s002.docx]

**S1 Text. ^1^H–^1^H gs-COSY and ^1^H–^13^C gs-HMQC spectra of complexes 3, 7 and 8.** The complexes were dissolved in DMF-*d_7_*.

^1^H–^1^H gs-COSY spectrum of complex *cis*-[Pt(*3Br*aza)_2_I_2_] (**3**)





^1^H–^13^C gs-HMQC spectrum of complex *cis*-[Pt(*3Br*aza)_2_I_2_] (**3**)





^1^H–^1^H gs-COSY spectrum of complex *cis*-[Pt(*5Br*aza)_2_I_2_] (**7**)


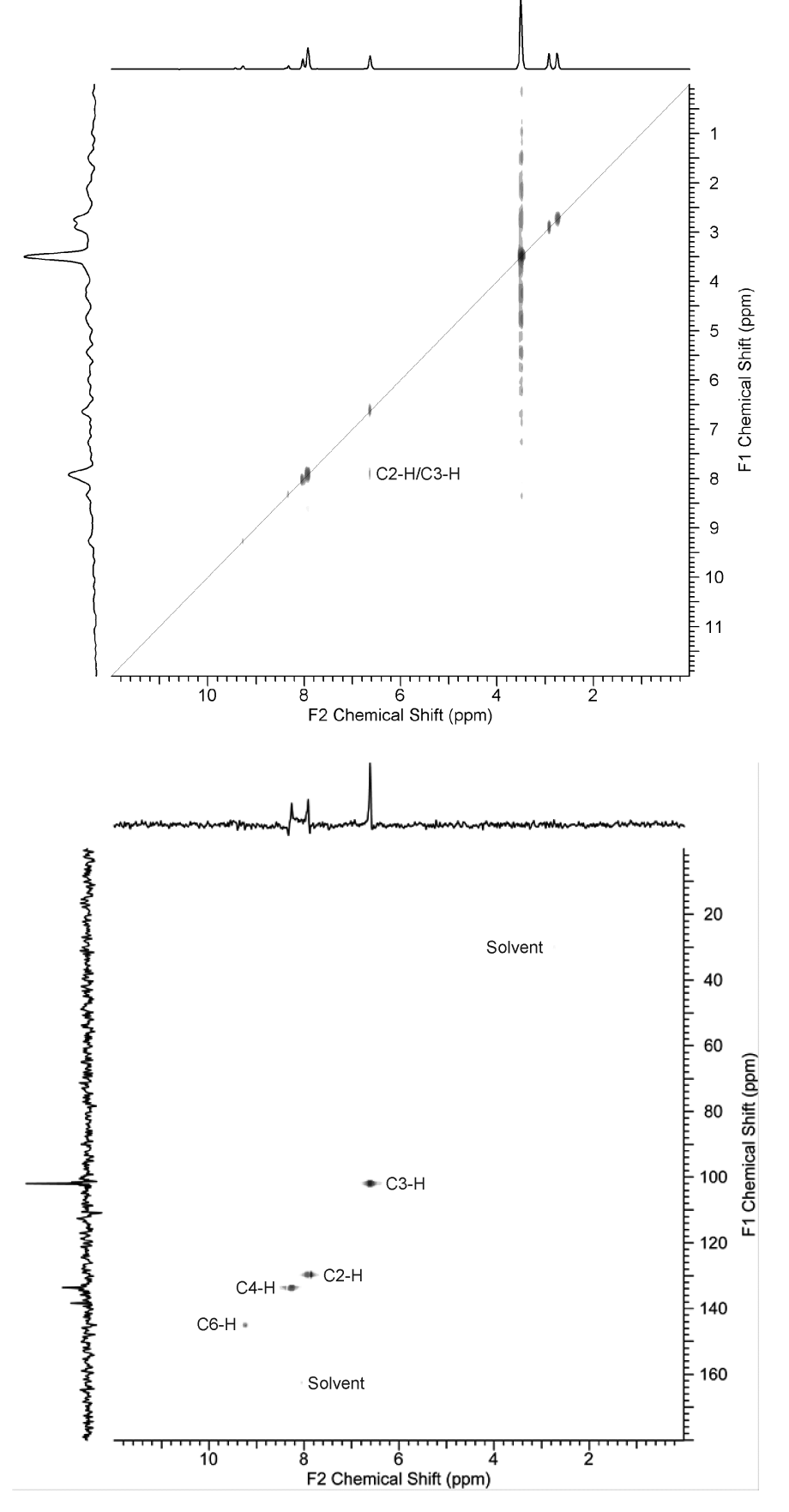


^1^H–^13^C gs-HMQC spectrum of complex *cis*-[Pt(*5Br*aza)_2_I_2_] (**7**)


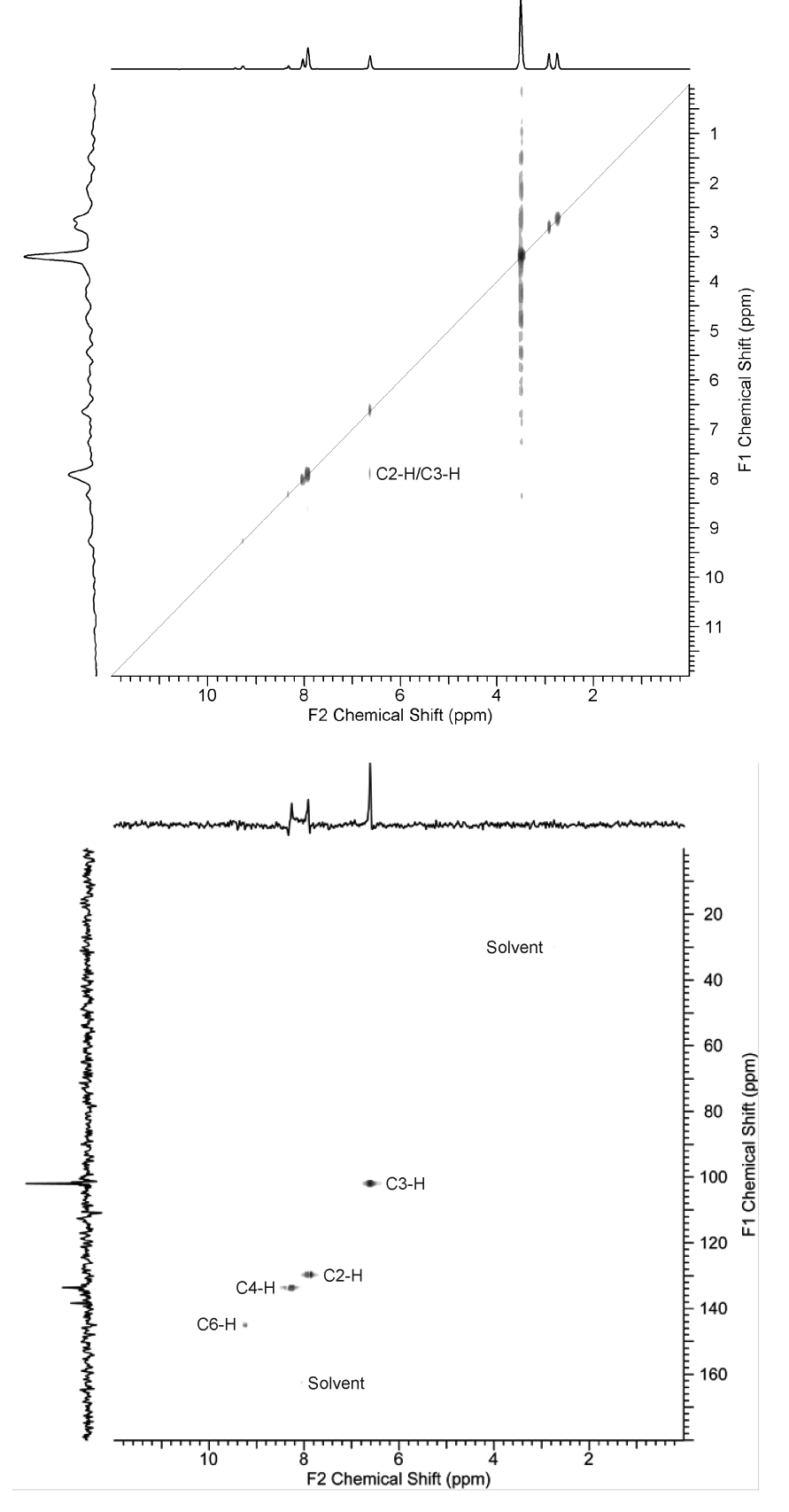


^1^H–^1^H gs-COSY spectrum of complex *cis*-[PtI_2_(*2Me4Cl*aza)_2_] (**8**)





^1^H–^13^C gs-HMQC spectrum of complex *cis*-[PtI_2_(*2Me4Cl*aza)_2_] (**8**)
