# Supplementary material for: Platinum(II) Iodido Complexes of 7-Azaindoles with Significant Antiproliferative Effects: An Old Story Revisited with Unexpected Outcomes
Source: PLoS One. 2016 Dec 1;11(12):e0165062. doi: 10.1371/journal.pone.0165062 (PMC5131915; doi:10.1371/journal.pone.0165062)
Supplement: S2 Table — (PDF) [file pone.0165062.s004.pdf]

S2 Table. The ^{1}H and ^{13}C NMR coordination shifts (calculated as $\Delta\delta = \delta_{complex} - \delta_{ligand}$; ppm) of the prepared complexes.

	^{1}H NMR						^{13}C NMR						
	N1H	C2H	C3H	C4H	C5H	C6H	C2	C3	C3a	C4	C5	C6	C7a
**1**	-	0.25	0.10	0.05	0.13	0.70	2.5	2.7	4.1	4.0	2.1	3.1	-0.5
**2**	-	0.30	-	0.11	0.17	0.75	2.8	2.3	4.4	4.2	2.0	3.2	-1.2
**3**	-	0.33	-	0.11	0.13	0.77	2.9	2.3	4.3	4.2	2.3	3.2	-1.8
**4**	-	0.28	-	0.05	0.13	0.75	2.6	2.5	4.2	4.1	2.0	1.6	-1.4
**5**	-	0.27	0.09	-	0.17	0.76	2.2	2.8	4.1	3.8	2.4	2.9	-1.8
**6**	-	0.26	0.08	-	0.16	0.75	2.2	2.8	4.1	4.3	2.3	2.6	-1.9
**7**	-	0.27	0.10	0.07	-	0.96	2.6	2.7	3.9	4.1	-0.2	2.9	-0.7
**8**	-	-	0.11	-	0.17	0.74	2.9	1.9	2.9	1.8	1.7	0.6	-2.5
