# Supplementary material for: Platinum(II) Iodido Complexes of 7-Azaindoles with Significant Antiproliferative Effects: An Old Story Revisited with Unexpected Outcomes
Source: PLoS One. 2016 Dec 1;11(12):e0165062. doi: 10.1371/journal.pone.0165062 (PMC5131915; doi:10.1371/journal.pone.0165062)
Supplement: S2 Text — (PDF) [file pone.0165062.s005.pdf]

S2 Text. ESI-MS data for complexes 1–8.

cis-[PtI$_2$(aza)$_2$] (**1**): ESI+ MS (MeOH): 723.8 (calc. 723.8 for {[PtI$_2$(aza)$_2$]+K}$^{+}$; 15 %), 707.9 (calc. 707.9 for {[PtI$_2$(aza)$_2$]+Na}$^{+}$; 100 %), 685.8 (calc. 685.9 for {[PtI$_2$(aza)$_2$]+H}$^{+}$; 5 %), 558.0 (calc. 558.0 for [PtI(aza)$_2$]$^{+}$; 15 %), 119.2 (calc. 119.1 for {(aza)+H}$^{+}$; 50 %) *m/z*. ESI– MS (MeOH): 683.8 (calc. 683.9 for {[PtI$_2$(aza)$_2$]–H}$^{-}$; 15 %), 566.1 (calc. 565.8 for {[PtI$_2$(aza)]–H}$^{-}$; 100 %), 575.8 (calc. 575.7 for [PtI$_3$]$^{-}$; 5 %), 127.2 (calc. 126.9 for {I}$^{-}$; 5 %) *m/z*.

cis-[PtI$_2$(*3Cl*aza)$_2$] (**2**): ESI+ MS (MeOH): 792.6 (calc. 792.8 for {[PtI$_2$(*3Cl*aza)$_2$]+K}$^{+}$; 80 %), 776.7 (calc. 776.8 for {[PtI$_2$(*3Cl*aza)$_2$]+Na}$^{+}$; 60 %), 626.9 (calc. 626.9 for [PtI(*3Cl*aza)$_2$]$^{+}$; 20 %), 153.0 (calc. 153.0 for {(*3Cl*aza)+H}$^{+}$; 100 %) *m/z*. ESI– MS (MeOH): 752.6 (calc. 752.8 for {[PtI$_2$(*3Cl*aza)$_2$]–H}$^{-}$; 20 %), 728.5 (calc. 728.7 for [PtI$_3$(*3Cl*aza)]$^{-}$; 5 %), 600.8 (calc. 600.8 for {[PtI$_2$(*3Cl*aza)]–H}$^{-}$; 100 %), 575.9 (calc. 575.7 for [PtI$_3$]$^{-}$; 30 %), 474.0 (calc. 473.9 for {[PtI(*3Cl*aza)]–H}$^{-}$; 25 %), 151.1 (calc. 151.0 for {(*3Cl*aza)–H}$^{-}$; 30 %), 127.0 (calc. 126.9 for {I}$^{-}$; 30 %) *m/z*.

cis-[PtI$_2$(*3Br*aza)$_2$] (**3**): ESI+ MS (MeOH): 882.6 (calc. 882.7 for {[PtI$_2$(*3Br*aza)$_2$]+K}$^{+}$; 20 %), 866.7 (calc. 866.7 for {[PtI$_2$(*3Br*aza)$_2$]+Na}$^{+}$; 60 %), 197.1 (calc. 197.0 for {(*3Br*aza)+H}$^{+}$; 100 %) *m/z*. ESI– MS (MeOH): 842.6 (calc. 842.7 for {[PtI$_2$(*3Br*aza)$_2$]–H}$^{-}$; 100 %), 772.7 (calc. 772.6 for [PtI$_3$(*3Br*aza)]$^{-}$; 10 %), 645.0 (calc. 644.7 for {[PtI$_2$(*3Br*aza)]–H}$^{-}$; 100 %), 576.0 (calc. 575.7 for [PtI$_3$]$^{-}$; 60 %), 518.2 (calc. 517.8 for {[PtI(*3Br*aza)]–H}$^{-}$; 25 %), 195.2 (calc. 195.0 for {(*3Br*aza)–H}$^{-}$; 5 %), 127.2 (calc. 126.9 for {I}$^{-}$; 5 %) *m/z*.

cis-[PtI$_2$(*3I*aza)$_2$] (**4**): ESI+ MS (MeOH): 959.6 (calc. 959.7 for {[PtI$_2$(*3I*aza)$_2$]+Na}$^{+}$; 95 %), 809.7 (calc. 809.8 for [PtI(*3I*aza)$_2$]$^{+}$; 5 %), 245.0 (calc. 245.0 for {(*3I*aza)+H}$^{+}$; 100 %) *m/z*. ESI– MS (MeOH): 935.5 (calc. 935.7 for {[PtI$_2$(*3I*aza)$_2$]–H}$^{-}$; 95 %), 819.5 (calc. 819.6 for [PtI$_3$(*3I*aza)]$^{-}$; 20 %), 691.9 (calc. 691.7 for {[PtI$_2$(*3I*aza)]–H}$^{-}$; 100 %), 575.9 (calc. 575.7 for [PtI$_3$]$^{-}$; 25 %), 565.0 (calc. 564.8 for {[PtI(*3I*aza)]–H}$^{-}$; 10 %), 243.1 (calc. 242.9 for {(*3I*aza)–H}$^{-}$; 10 %), 127.0 (calc. 126.9 for {I}$^{-}$; 5 %) *m/z*.

cis-[PtI$_2$(*4Cl*aza)$_2$] (**5**): ESI+ MS (MeOH): 776.8 (calc. 776.8 for {[PtI$_2$(*4Cl*aza)$_2$]+Na}$^{+}$; 100 %), 626.9 (calc. 626.9 for [PtI(*4Cl*aza)$_2$]$^{+}$; 5 %), 153.1 (calc. 153.0 for {(*4Cl*aza)+H}$^{+}$; 20 %) *m/z*. ESI– MS (MeOH): 752.7 (calc. 752.8 for {[PtI$_2$(*4Cl*aza)$_2$]–H}$^{-}$; 100 %), 728.6 (calc. 728.7 for [PtI$_3$(*4Cl*aza)]$^{-}$; 10 %), 601.0 (calc. 600.8 for {[PtI$_2$(*4Cl*aza)]–H}$^{-}$; 60 %), 575.9 (calc. 575.7 for [PtI$_3$]$^{-}$; 15 %), 151.1 (calc. 151.0 for {(*4Cl*aza)–H}$^{-}$; 5 %), 127.0 (calc. 126.9 for {I}$^{-}$; 5 %) *m/z*.

cis-[PtI_2(*4Br*aza)$_2$] (**6**): ESI+ MS (MeOH): 882.6 (calc. 882.7 for {[PtI_2(*4Br*aza)$_2$]+K}$^+$; 10 %), 866.6 (calc. 866.7 for {[PtI_2(*4Br*aza)$_2$]+Na}$^+$; 100 %), 716.8 (calc. 716.8 for [PtI(*4Br*aza)$_2$]$^+$; 5 %), 197.0 (calc. 197.0 for {(*4Br*aza)+H}$^+$; 70 %) *m/z*. ESI– MS (MeOH): 842.5 (calc. 842.7 for {[PtI_2(*4Br*aza)$_2$]–H}$^-$; 100 %), 772.6 (calc. 772.6 for [PtI_3(*4Br*aza)]$^-$; 25 %), 644.9 (calc. 644.7 for {[PtI_2(*4Br*aza)]–H}$^-$; 85 %), 575.9 (calc. 575.7 for [PtI_3]$^-$; 35 %), 518.0 (calc. 517.8 for {[PtI(*4Br*aza)]–H}$^-$; 5 %), 195.1 (calc. 195.0 for {(*4Br*aza)–H}$^-$; 5 %), 126.9 (calc. 126.9 for {I}$^-$; 30 %) *m/z*.

cis-[PtI_2(*5Br*aza)$_2$] (**7**): ESI+ MS (MeOH): 866.6 (calc. 866.7 for {[PtI_2(*5Br*aza)$_2$]+Na}$^+$; 100 %), 716.8 (calc. 716.8 for [PtI(*5Br*aza)$_2$]$^+$; 5 %), 197.0 (calc. 197.0 for {(*5Br*aza)+H}$^+$; 10 %) *m/z*. ESI– MS (MeOH): 842.5 (calc. 842.7 for {[PtI_2(*5Br*aza)$_2$]–H}$^-$; 100 %), 772.6 (calc. 772.6 for [PtI_3(*5Br*aza)]$^-$; 15 %), 644.9 (calc. 644.7 for {[PtI_2(*5Br*aza)]–H}$^-$; 95 %), 575.9 (calc. 575.7 for [PtI_3]$^-$; 20 %), 195.1 (calc. 195.0 for {(*5Br*aza)–H}$^-$; 10 %), 127.0 (calc. 126.9 for {I}$^-$; 5 %) *m/z*.

cis-[PtI_2(*2Me4Cl*aza)$_2$] (**8**): ESI-MS (MeOH): 820.9 (calc. 820.8 for {[PtI_2(*2Me4Cl*aza)$_2$]+K}$^+$; 50 %), 804.8 (calc. 804.8 for {[PtI_2(*2Me4Cl*aza)$_2$]+Na}$^+$; 10 %), 654.9 (calc. 654.9 for [PtI(*2Me4Cl*aza)$_2$]$^+$; 10 %), 167.1 (calc. 167.0 for {(*2Me4Cl*aza)+H}$^+$; 100 %) *m/z*. ESI– MS (MeOH): 780.9 (calc. 780.8 for {[PtI_2(*2Me4Cl*aza)$_2$]–H}$^-$; 100 %), 615.1 (calc. 614.8 for {[PtI_2(*4Br*aza)]–H}$^-$; 90 %), 576.1 (calc. 575.7 for [PtI_3]$^-$; 20 %), 487.2 (calc. 487.9 for {[PtI(*4Br*aza)]–H}$^-$; 5 %), 127.1 (calc. 126.9 for {I}$^-$; 5 %) *m/z*.
