# Supplementary figures and images for: Platinum(II) Iodido Complexes of 7-Azaindoles with Significant Antiproliferative Effects: An Old Story Revisited with Unexpected Outcomes
Source: PLoS One. 2016 Dec 1;11(12):e0165062. doi: 10.1371/journal.pone.0165062 (PMC5131915; doi:10.1371/journal.pone.0165062)

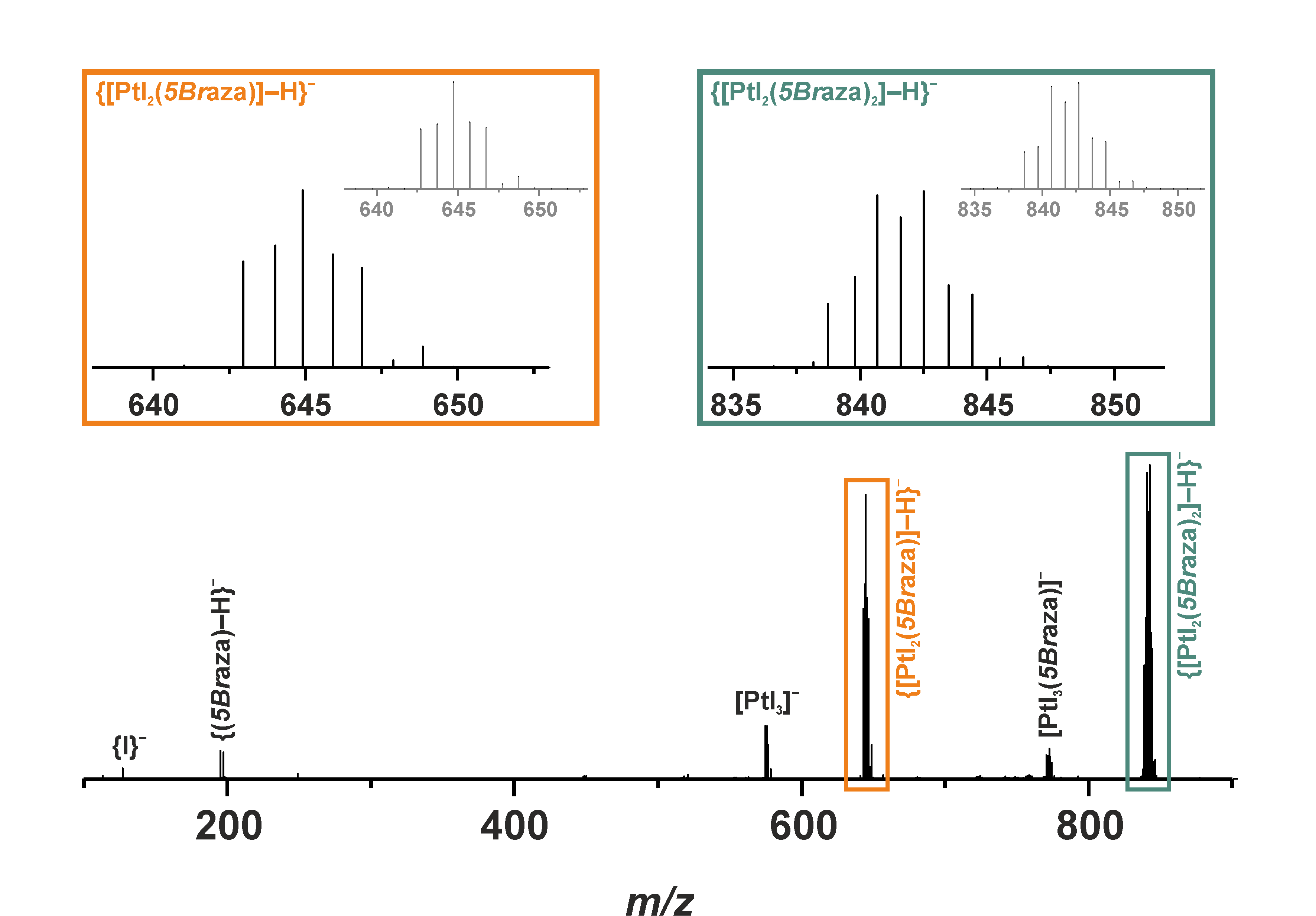

Supplement: S1 Fig — The complex was dissolved in methanol and an assignment of the observed peaks and details of experimental (black) and simulated (grey) isotopic distribution of the {[PtI2(5Braza)2]–H}−(green) and {[PtI2(5Braza)]–H}−(orange) species is included. (TIF) [file pone.0165062.s006.tif]

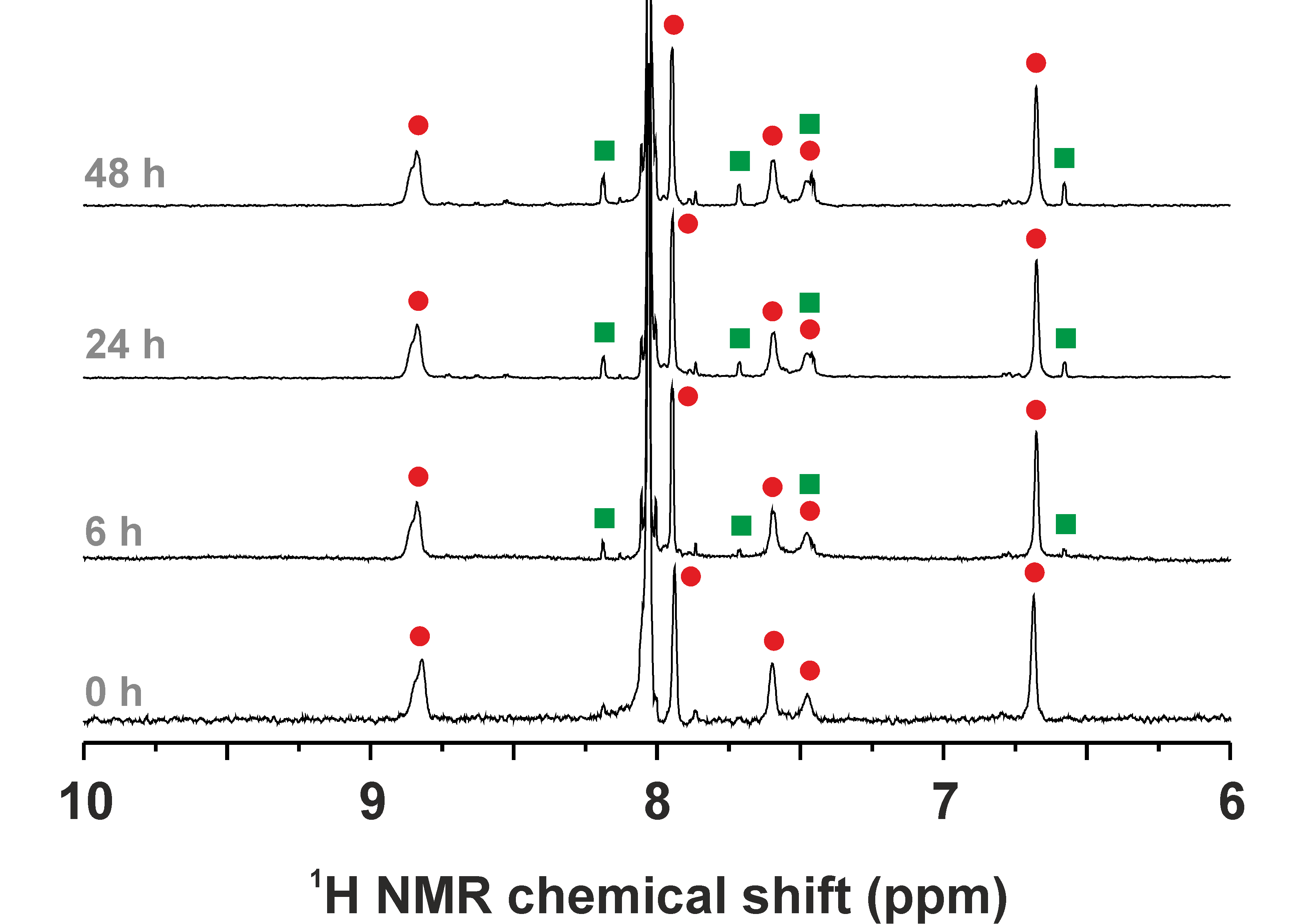

Supplement: S2 Fig — The signals of complex 6 are marked with circles and the signals of the hydrolysed form of complex 6 are marked with squares. (TIF) [file pone.0165062.s007.tif]

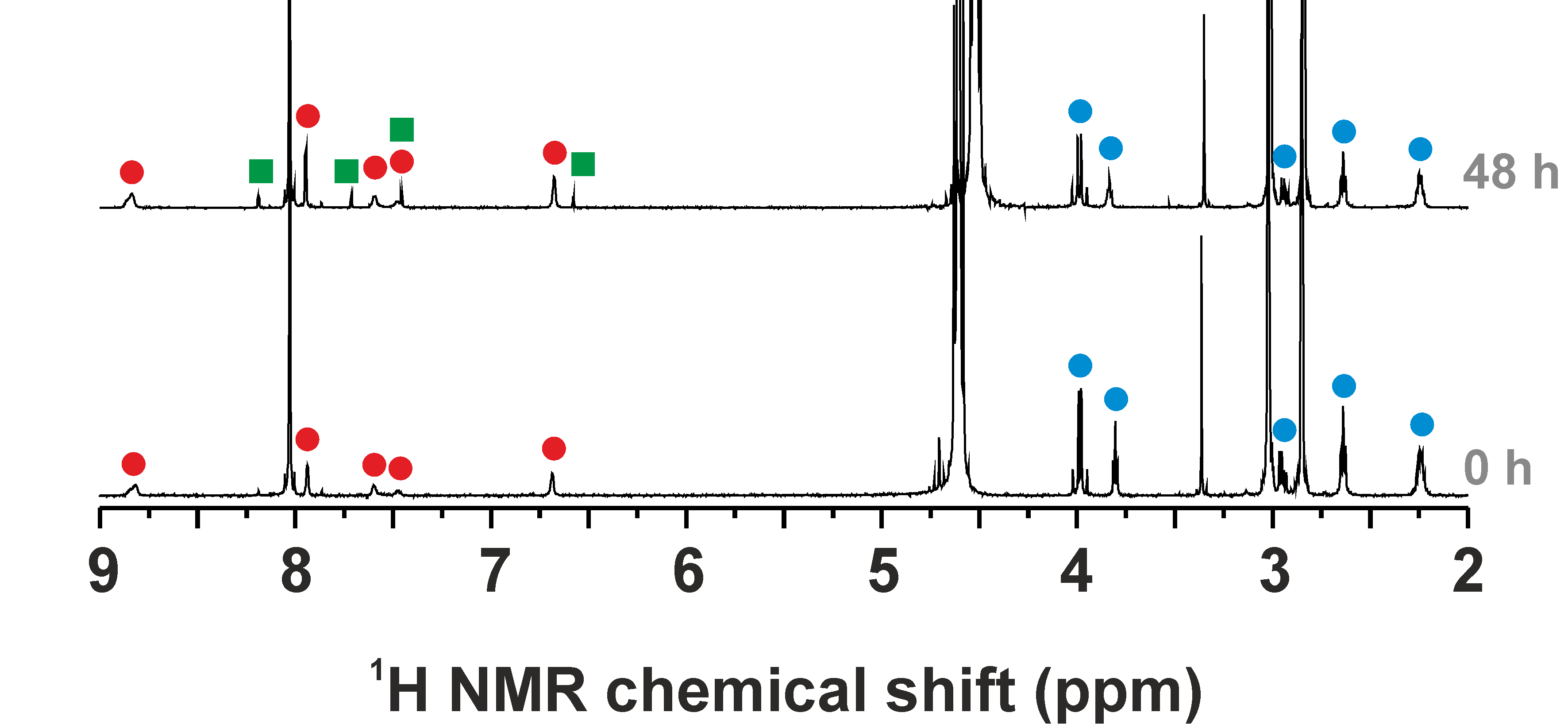

Supplement: S3 Fig — The signals of complex 6 are marked with red circles, the signals of the hydrolysed form of complex 6 are marked with green squares and blue circles mark the signals of GSH. (TIF) [file pone.0165062.s008.tif]

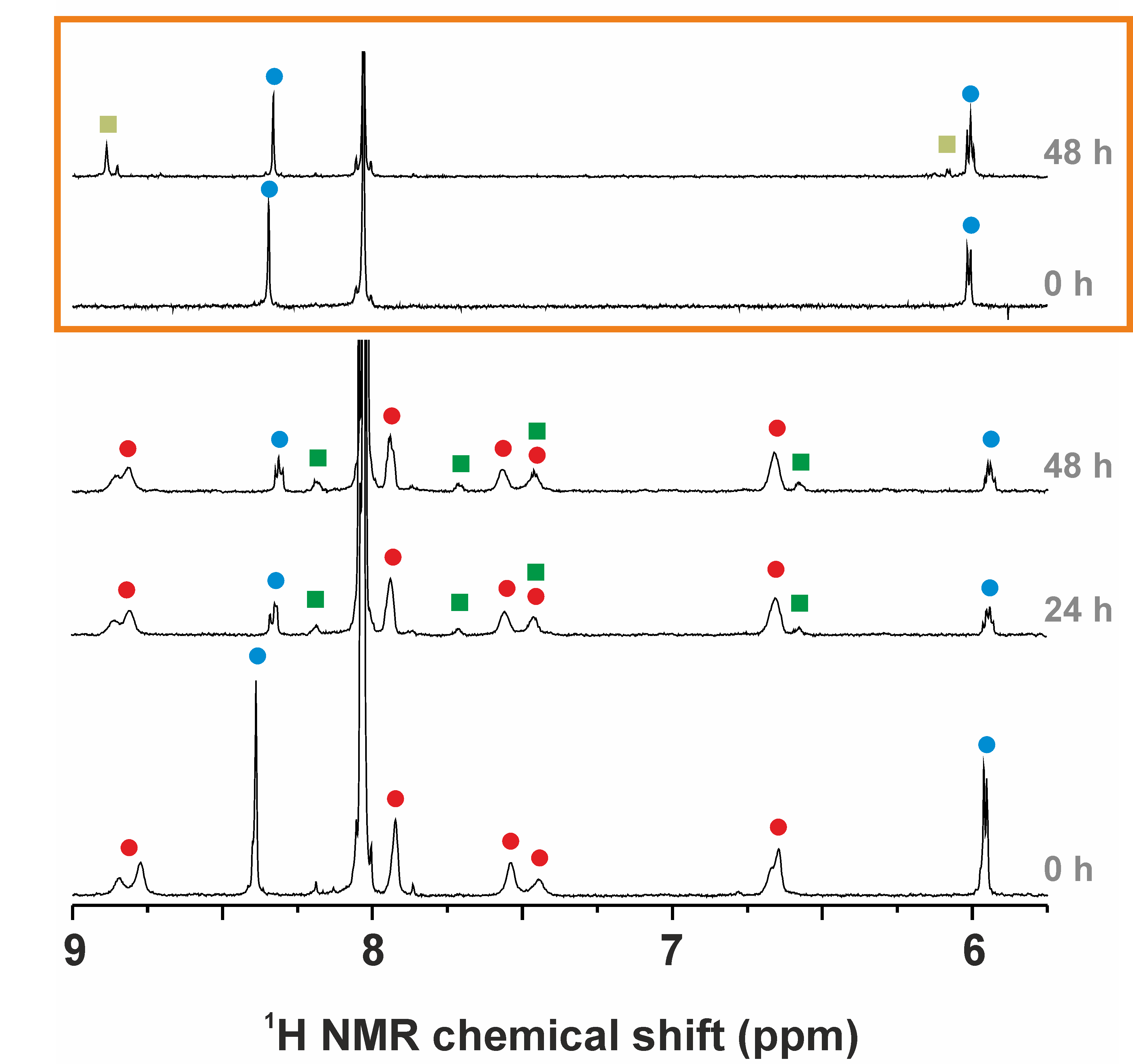

Supplement: S4 Fig — The signals of complex 6 are marked with red circles, the signals of the hydrolysed form of complex 6 are marked with green squares, blue circles mark the signals of GMP and yellow squares assign Pt–GMP adduct. (TIF) [file pone.0165062.s009.tif]

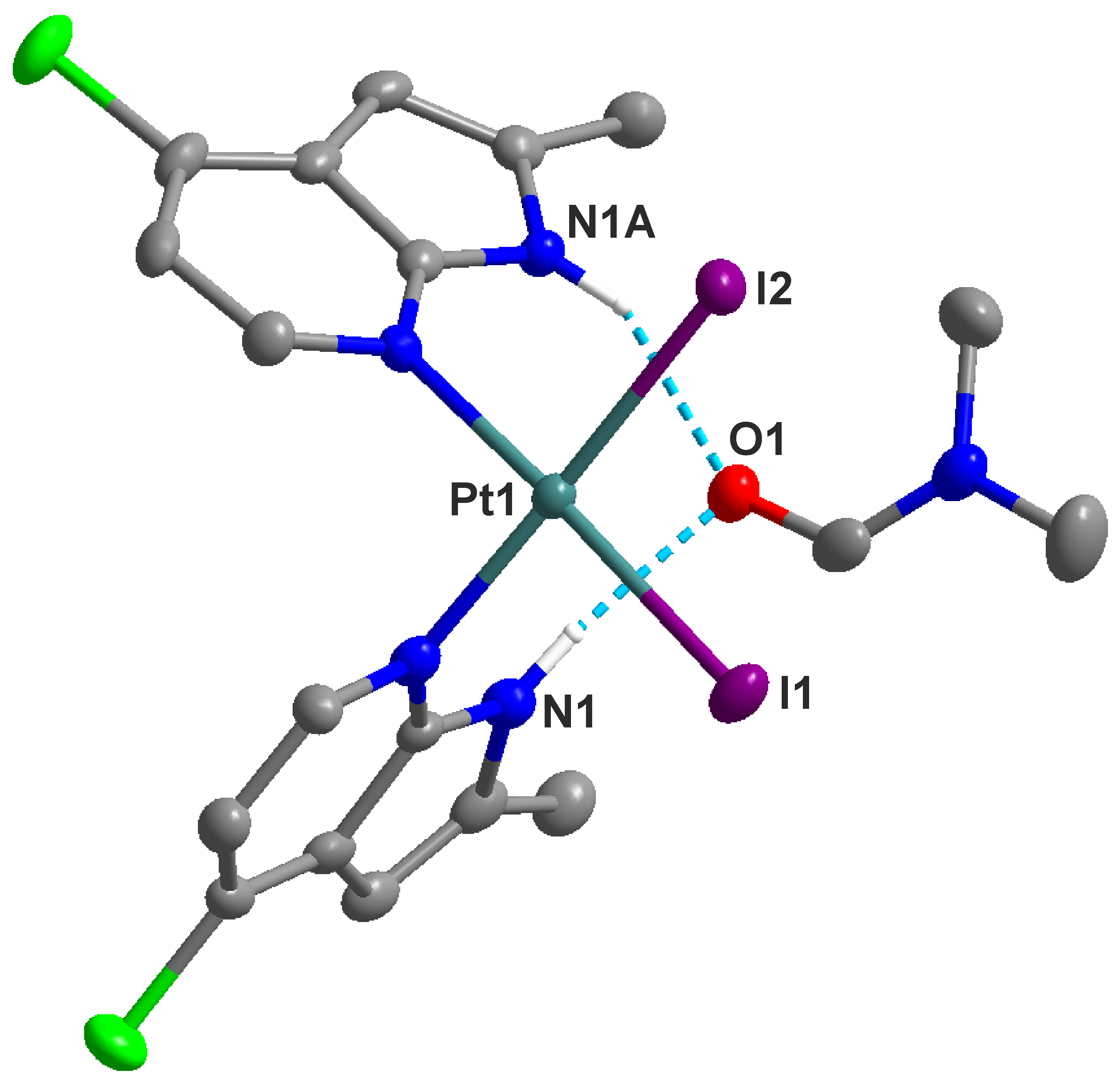

Supplement: S5 Fig — The hydrogen atoms not involved in the depicted hydrogen bonds were omitted for clarity. (TIF) [file pone.0165062.s010.tif]

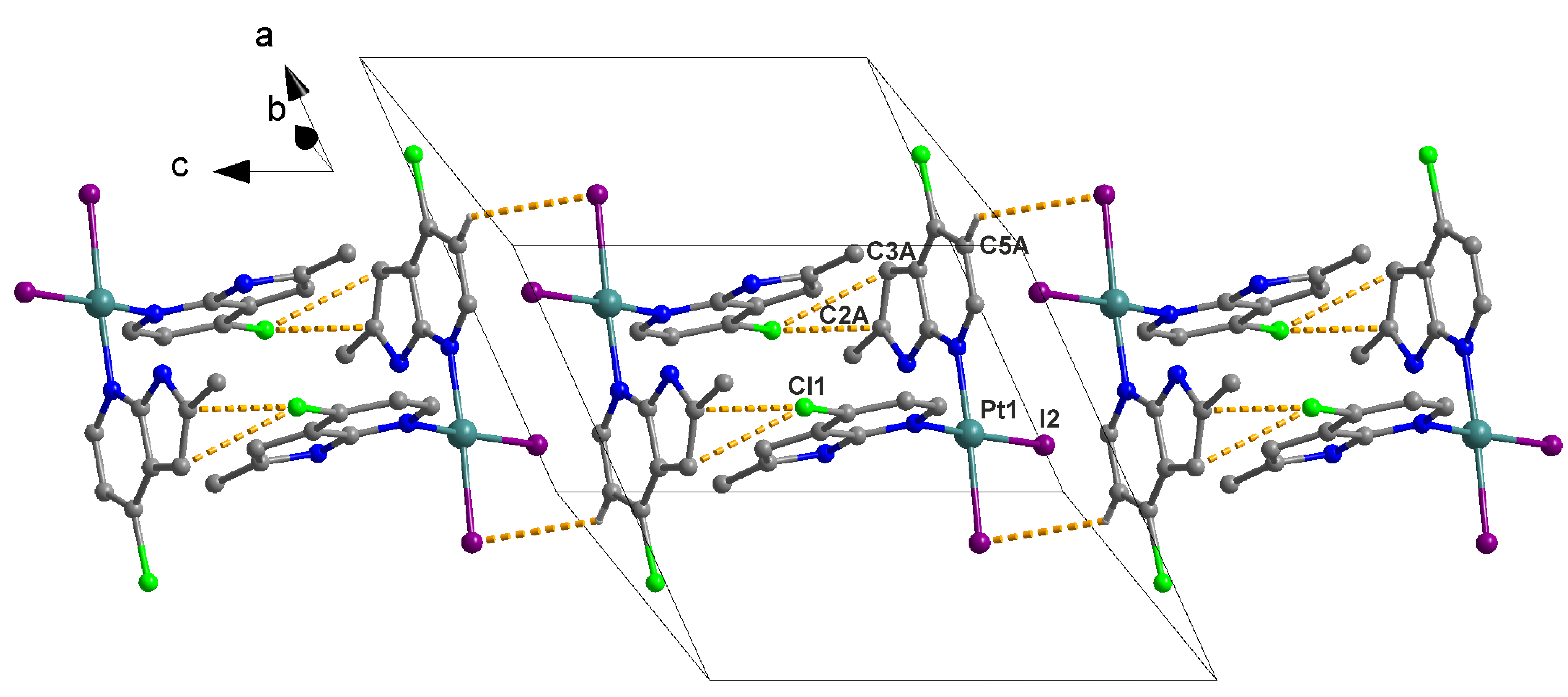

Supplement: S6 Fig — The hydrogen atoms not involved in the depicted non-covalent contacts and DMF molecule of crystallization were omitted for clarity. (TIF) [file pone.0165062.s012.tif]

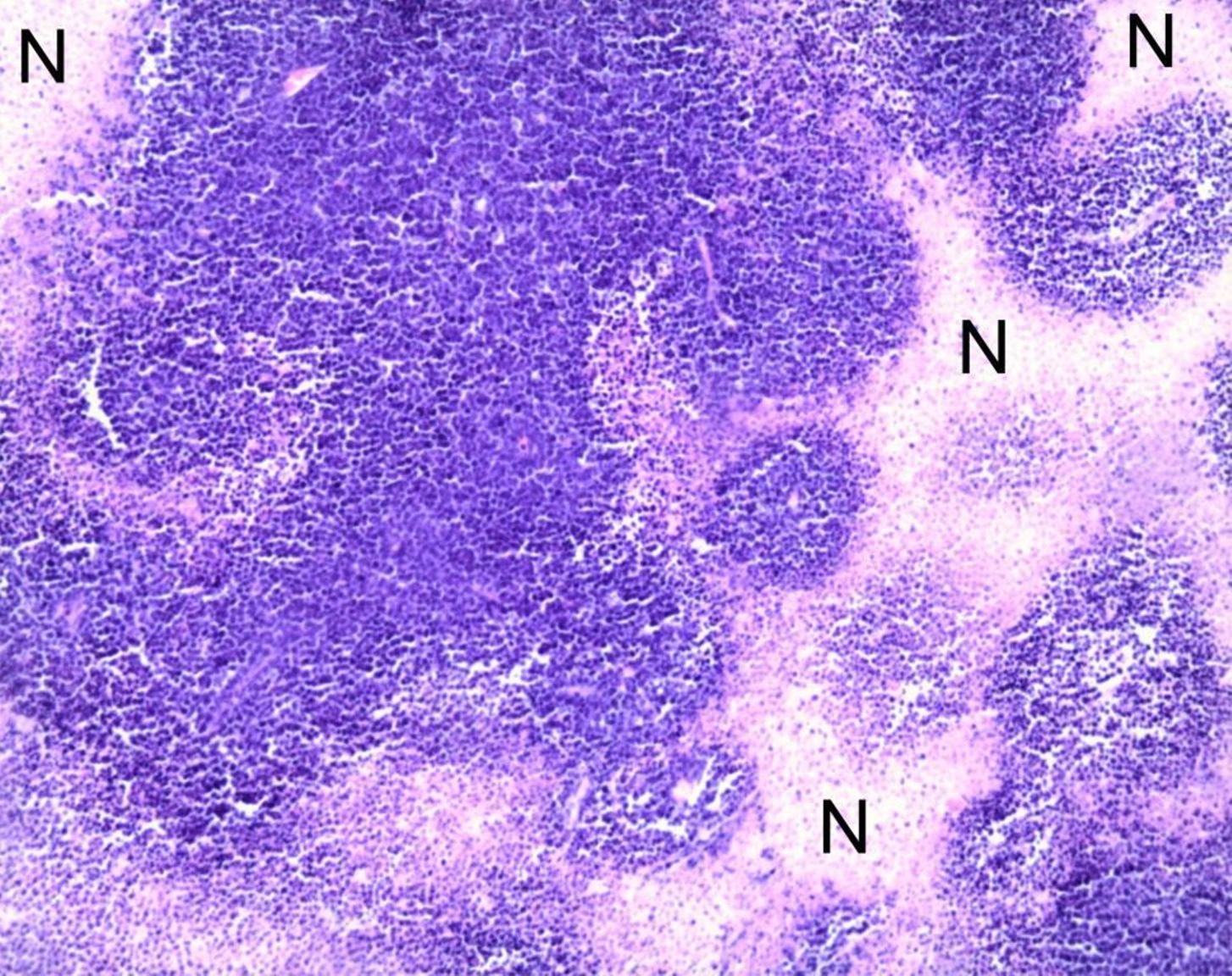

Supplement: S7 Fig — The tissue sample was stained by standard hematoxylin and eosin staining and photographed at 100× magnification. (JPG) [file pone.0165062.s015.jpg]
