# Supplementary material for: Platinum(II) Iodido Complexes of 7-Azaindoles with Significant Antiproliferative Effects: An Old Story Revisited with Unexpected Outcomes
Source: PLoS One. 2016 Dec 1;11(12):e0165062. doi: 10.1371/journal.pone.0165062 (PMC5131915; doi:10.1371/journal.pone.0165062)
Supplement: S3 Table — (PDF) [file pone.0165062.s011.pdf]

S3 Table. The parameters of the non-covalent contacts in the crystal structure of *cis*-[PtI$_2$(*2Me4Cl*aza)$_2$]·DMF (8·DMF).

D−H···A	*d*(D···A)	< (DHA)	Symmetry code
N1–H1A···O1	2.841(6)	171.6	-
N1A–H11B···O1	2.877(6)	142.6	-
C2A···Cl1^{i}	3.142(6)	-	1-x, 1-y, 1-z
C3A···Cl1^{i}	3.354(5)	-	1-x, 1-y, 1-z
C5A–H51A···I1^{ii}	3.816(5)	129.2	1-x, 1-y, -z
C6–H6A···I2^{ii}	3.892(5)	126.3	1-x, 1-y, -z
C8–H8B···I1^{iii}	3.818(6)	128.0	-x, 1-y, 1-z
C8A–H81B···C11^{iv}	3.614(7)	132.3(3)	-x, 2-y, 1-z
C9–H9A···C3A^{iii}	3.536(10)	127.9(5)	-x, 1-y, 1-z
C10–H10C···I2	4.002(7)	144.0	-
C11–H11A···I1	3.629(5)	110.1	-
