# Supplementary material for: Platinum(II) Iodido Complexes of 7-Azaindoles with Significant Antiproliferative Effects: An Old Story Revisited with Unexpected Outcomes
Source: PLoS One. 2016 Dec 1;11(12):e0165062. doi: 10.1371/journal.pone.0165062 (PMC5131915; doi:10.1371/journal.pone.0165062)
Supplement: S4 Table — (PDF) [file pone.0165062.s013.pdf]

S4 Table. The resistance factors, defined as the ratio between *in vitro* cytotoxicity (IC_{50}) against resistant and sensitive variants of the A2780 ovarian cancer cell line (IC_{50}-A2780R/IC_{50}-A2780), calculated for complexes 1–8 and *cisplatin*.

**1**	**2**	**3**	**4**	**5**	**6**	**7**	**8**	*Cisplatin*
0.94	0.89	1.14	1.13	1.10	1.03	1.03	0.59	>1.78
