# Supplementary material for: Platinum(II) Iodido Complexes of 7-Azaindoles with Significant Antiproliferative Effects: An Old Story Revisited with Unexpected Outcomes
Source: PLoS One. 2016 Dec 1;11(12):e0165062. doi: 10.1371/journal.pone.0165062 (PMC5131915; doi:10.1371/journal.pone.0165062)
Supplement: S5 Table — The experimental conditions were as follows 24 h exposure time; IC50 concentrations applied and stained with PI/RNase. The data are given as arithmetic mean±SD from three independent experiments. (PDF) [file pone.0165062.s014.pdf]

S5 Table. The sub-G1, G0/G1, S and G2/M populations (%) detected at MCF7 and A2780 human cancer cell lines treated with complexes 6 and 8, and *cisplatin* for comparative purposes. The experimental conditions were as follows 24 h exposure time; IC_{50} concentrations applied and stained with PI/RNase. The data are given as arithmetic mean±SD from three independent experiments.

Cell Cycle Phase	**6**		**8**		*Cisplatin*		Control	
	MCF-7	A2780	MCF-7	A2780	MCF-7	A2780	MCF-7	A2780
sub-G1	1.5±0.4	4.3±1.4	1.2±0.4	2.2±0.4	1.6±0.2	2.0±0.2	-	-
G0/G1	73.9±4.6	51.5±3.2	64.5±3.3	55.2±2.2	33.4±2.8	23.7±1.5	65.3±2.9	65.1±4.1
S	7.2±1.6	17.5±0.9	8.9±2.1	18.3±1.3	26.3±1.9	50.6±2.8	10.7±1.3	16.0±1.2
G2/M	16.6±2.1	26.3±1.6	25.4±2.6	23.9±2.3	38.2±1.9	23.0±2.2	23.7±1.6	17.8±1.3
